# From laparoscopy to robotics in living donor hepatectomy: a systematic review and meta-analysis of comparative outcomes

**DOI:** 10.1007/s11701-026-03360-2

**Published:** 2026-05-14

**Authors:** Mohamed Al Sayed, Mahmoud Albashier, Hashem Altabbaa, Ahmad Omar Saleh, Abdalla M. Hadhoud, Mahmoud Ashraf Hussein, Abedalrahman Yazan Aljarrah, Menatalla M. Abdelrazek, Mohamed Bakhiet

**Affiliations:** 1 Department of Upper Gastrointestinal and Liver Surgery, Unit 2, Alexandria Main University Hospital, Alexandria University, Alexandria, Egypt; 2 Department of Surgery, Faculty of Medicine, Alexandria University, Alexandria, Egypt; 3 The Research Papyrus Lab, Alexandria, Egypt; 4 Faculty of Medicine, Damietta University, New Damietta, Egypt; 5 Al-Basheer-Hospital, Amman, Jordan; 6 Faculty of Medicine, The University of Jordan, Amman, Jordan; 7 Department of Surgery, University of Cincinnati, Cincinnati, OH USA; 8 Faculty of Medicine, Capital University, Cairo, Egypt; 9 Princess Basma Teaching Hospital, Ministry of Health, Irbid, Jordan; 10 Alexandria Main University Hospital, Alexandria University, Alexandria, Egypt; 11 School of Medicine, University of Aberdeen, Aberdeen, UK

**Keywords:** Adult living donor liver transplantation, Robotic living donor hepatectomy, Laparoscopic donor hepatectomy, Minimally invasive liver surgery, Donor safety, Meta-analysis

## Abstract

Minimally invasive approaches have become integral to adult living donor hepatectomy, where protection of donor safety is paramount. Both laparoscopic and robotic techniques are increasingly implemented in specialized transplant centers for various graft types, including right lobe, left lobe, and left lateral sectionectomy. In this context, we conducted a systematic review and meta-analysis to synthesize the current evidence comparing robotic and laparoscopic living donor hepatectomy in adult liver transplantation. The analysis primarily focused on right lobe procurement while acknowledging that several large registry-based studies included mixed graft types. The methodology and reporting of this study followed the PRISMA 2020 statement, and the protocol was prospectively registered in the PROSPERO database. Electronic databases were systematically searched through December 2025 to identify comparative studies evaluating robotic and laparoscopic hepatectomy in adult living donors. Operative, donor, and recipient outcomes were synthesized using random-effects meta-analytic models. Methodological quality was appraised with the ROBINS-I tool, and the overall strength of evidence was graded using the GRADE framework. Seven comparative studies published between 2022 and 2025, comprising more than 15,000 adult living donors, met inclusion criteria. Baseline demographic and clinical characteristics were broadly similar across surgical approaches. Robotic donor hepatectomy was associated with a lower likelihood of perioperative transfusion (RR 0.27, 95% CI 0.09–0.76; p = 0.013) and a reduced incidence of overall donor morbidity (RR 0.47, 95% CI 0.25–0.89; p = 0.020) compared with laparoscopy. No significant differences were observed in major donor complications or mortality. Although operative duration was longer with the robotic technique, intraoperative blood loss was lower. Among recipients, robotic graft procurement was associated with reduced overall morbidity and fewer biliary complications, whereas vascular events and mortality were comparable between approaches. Robotic living donor hepatectomy appears to enhance donor safety, with lower transfusion rates and reduced overall morbidity, while maintaining comparable rates of major complications and mortality. These results suggest a potential advantage for the robotic approach in appropriately experienced centers. Nevertheless, confirmation through large, rigorously designed randomized trials is required to establish definitive comparative effectiveness and to clarify the optimal minimally invasive strategy.

**Registration: **PROSPERO CRD420261299554.

**Supplementary Information:**

The online version contains supplementary material available at 10.1007/s11701-026-03360-2.

## Introduction

The adoption of minimally invasive techniques has fundamentally altered the landscape of adult living donor hepatectomy (LDH), where safeguarding donor well-being remains paramount while ensuring uncompromised graft function. Since laparoscopic living donor hepatectomy was first described in 2002 [1], selected transplant centers have progressively integrated minimally invasive strategies into donor surgery. Accumulating observational evidence and several systematic evaluations have demonstrated that, compared with the conventional open approach, minimally invasive donor hepatectomy is associated with reduced perioperative morbidity, shorter hospitalization, and improved postoperative recovery, without jeopardizing graft integrity or recipient outcomes [2–4].

The introduction of robotic assistance in 2012 marked another milestone in the evolution of donor hepatectomy [5]. Robotic systems provide enhanced depth perception, motion scaling, tremor suppression, and wristed instrumentation, potentially improving precision during parenchymal transection and hilar dissection [5, 6]. These technical attributes have encouraged increasing adoption of robotic donor hepatectomy, particularly in centers with established expertise in complex hepatobiliary and transplant procedures [7].

Although both laparoscopic and robotic approaches are now performed in specialized programs, most published comparative studies have evaluated minimally invasive techniques collectively against open surgery, rather than examining differences between the two minimally invasive modalities themselves [8, 9]. Consequently, direct comparative evidence assessing donor safety and recipient outcomes between laparoscopic living donor hepatectomy (L-LDH) and robotic living donor hepatectomy (R-LDH) remains relatively limited. Only in recent years have propensity score–matched analyses begun to offer head-to-head comparisons, highlighting the need for a structured and comprehensive synthesis of available data [10].

To address this evidence gap, we undertook a systematic review with meta-analytic synthesis of comparative studies evaluating robotic and laparoscopic living donor hepatectomy in adults, in accordance with PRISMA guidance. The analysis focused on operative performance, donor perioperative morbidity and mortality, graft ischemic variables, and recipient outcomes. By integrating currently available comparative data, this investigation seeks to delineate the relative advantages and trade-offs of each minimally invasive strategy and to inform clinical decision-making in adult living donor liver transplantation.

## Methods

### Study protocol and registration

The design and reporting of this review followed the 2020 update of the PRISMA statement [11], and methodological decisions were guided by recommendations outlined in the Cochrane Handbook for Systematic Reviews of Interventions [12]. Prior to data extraction, the study protocol was prospectively registered in the PROSPERO database (CRD420261299554) to ensure transparency and methodological consistency [13].

### Eligibility criteria

We included comparative studies evaluating adult living donors (aged ≥ 18 years) undergoing hepatectomy for liver transplantation in which robotic-assisted and laparoscopic donor hepatectomy were directly compared. Studies reporting outcomes for right lobe procurement were eligible for inclusion. Large registry studies and multicenter collaborations that included mixed graft types (right lobe, left lobe, and left lateral sectionectomy) were also retained to maximize sample size and enhance generalizability, provided that comparative outcomes between robotic and laparoscopic approaches were reported. To address potential heterogeneity related to graft type, sensitivity analyses restricted to studies exclusively reporting right lobe hepatectomy were conducted when data were available.

To qualify for inclusion, studies were required to report at least one clinically relevant perioperative or postoperative outcome. Outcomes of interest encompassed intraoperative parameters (operative time, estimated blood loss, conversion to open surgery), postoperative recovery metrics (length of hospital stay), and safety endpoints such as overall complications, major morbidity defined according to the Clavien–Dindo classification (grade ≥ III) [14], biliary and vascular events, reoperation, and donor mortality. When available, adult’s recipient outcomes attributable to graft procurement technique were also extracted. Both randomized controlled trials and observational comparative designs—including prospective, retrospective, and propensity score–matched cohort studies—were deemed eligible.

Publications were excluded if they lacked a comparator group, focused exclusively on pediatric donors, or represented case reports, case series without comparative analysis, narrative reviews, editorials, or conference abstracts with incomplete datasets. Non-English language articles and experimental (non-human) studies were also excluded. In instances where multiple reports described overlapping patient cohorts, the most comprehensive or most recently published dataset was retained to avoid duplication.

### Information sources

To ensure comprehensive coverage of the available literature, we systematically interrogated multiple electronic databases, including PubMed, Embase, Scopus, Web of Science, and the Cochrane Central Register of Controlled Trials (CENTRAL), from their inception through December 22, 2025. To minimize the risk of missing eligible studies, backward citation tracking was performed by screening the reference lists of all included articles as well as relevant review publications. Additionally, ClinicalTrials.gov was examined to identify ongoing or completed comparative studies evaluating robotic and laparoscopic living donor hepatectomy. Full-text versions of potentially eligible records were retrieved for detailed assessment whenever accessible. Only peer-reviewed articles published in English were considered for inclusion.

### Search strategy

The search approach was constructed to maximize sensitivity by combining controlled indexing terms with unrestricted keyword searches related to robotic and laparoscopic living donor hepatectomy in the context of liver transplantation. Database-specific subject headings—including Medical Subject Headings (MeSH) in PubMed and Emtree terms in Embase—were integrated with relevant synonyms, alternative spellings, and commonly used terminology (e.g., robotic hepatectomy, laparoscopic donor hepatectomy, living donor liver transplantation). Search expressions were adapted to the syntax requirements of each database and structured using Boolean logic to appropriately link concepts. The complete search algorithms, including all applied filters and limits, are provided in **Electronic Supplementary Material (ESM) 1** to facilitate transparency and reproducibility.

### Study selection

All records retrieved from the electronic searches were first consolidated within EndNote (version 20), where duplicate entries were identified and removed through combined automated and manual verification. The de-duplicated dataset was subsequently transferred to Rayyan to facilitate structured screening. Title and abstract evaluation was performed independently by two reviewers to determine potential eligibility. Articles considered potentially relevant by either reviewer underwent full-text assessment. Any disagreements arising during screening or eligibility evaluation were resolved through discussion, with arbitration by a senior investigator when consensus could not be reached. Eligibility of full-text reports was determined according to the predefined inclusion and exclusion criteria, and reasons for exclusion were systematically recorded.

### Data extraction

A structured data collection sheet was developed in Microsoft Excel to ensure uniform capture of relevant variables across studies. Extracted information was organized into three conceptual domains. First, general study descriptors were recorded, including author identification, country of origin, study design, sample size within each comparison arm, surgical technique, robotic platform (when specified), graft type, transplant indication, and reported duration of follow-up. Second, baseline demographic and clinical characteristics of donors and recipients were collected. These included group-specific sample sizes, donor age, sex distribution, and body mass index, as well as recipient age, sex, body mass index, Model for End-Stage Liver Disease (MELD) score, and graft-to-recipient weight ratio (GRWR). Continuous variables were extracted as means with standard deviations when available, while categorical variables were recorded as counts and proportions. Third, perioperative and postoperative endpoints predefined in the study protocol were retrieved. These encompassed operative metrics (e.g., operative duration, estimated blood loss, and conversion to open surgery), recovery indicators (length of hospital stay), and safety outcomes, including overall and major complications, biliary and vascular events, reintervention rates, donor mortality, and graft-related recipient outcomes when reported. Data extraction was performed independently by two reviewers to enhance reliability. Donor and recipient outcomes were analyzed separately, reflecting the fundamentally different operative procedures and mechanisms of complications in the donor hepatectomy and recipient implantation phases. The compiled datasets were subsequently compared to verify consistency, and disagreements were resolved through discussion, with involvement of a senior investigator if required. When reporting was incomplete or ambiguous, full manuscripts were carefully re-examined to clarify data. Where appropriate, additional statistics were calculated from available numerical information; outcomes that could not be reliably derived were excluded from quantitative pooling. To prevent duplication of patient populations, study characteristics and enrollment timeframes were cross-checked across publications. In cases of overlapping cohorts, preference was given to the most comprehensive or most recent report.

### Risk of bias assessment

Given that all included studies were observational in design, methodological quality was appraised at the study level using the ROBINS-I (Risk Of Bias In Non-randomized Studies of Interventions) instrument [15]. Two reviewers independently performed the evaluation. The ROBINS-I framework examines potential bias across seven predefined domains, including confounding, participant selection, intervention classification, deviations from intended interventions, incomplete outcome data, outcome measurement, and selective reporting. For each domain, studies were categorized as having low, moderate, serious, or critical risk of bias. These domain-level judgments were then synthesized to derive an overall risk-of-bias rating for each study. To facilitate transparent reporting, graphical representations summarizing domain-specific assessments were generated using the RobVis visualization tool [16]. Disagreements between assessors were resolved through discussion, and when necessary, a senior investigator provided arbitration to reach a final determination.

### Certainty of evidence (GRADE)

The overall strength of evidence for each analyzed outcome was appraised using the GRADE (Grading of Recommendations Assessment, Development, and Evaluation) framework [17, 18]. Assessments were performed independently by two reviewers. In accordance with GRADE principles, randomized studies were initially considered to provide high-certainty evidence, whereas observational studies began at a lower certainty level. The certainty rating for each outcome was then adjusted based on predefined criteria, including methodological limitations, variability across study results, indirectness of evidence, imprecision of pooled estimates, and potential reporting bias. Where concerns were identified in these domains, the certainty rating was downgraded accordingly. Following domain evaluation, outcomes were assigned one of four levels of certainty: very low, low, moderate, or high. Differences in judgment between reviewers were addressed through discussion until agreement was achieved. Summary of findings tables were generated using GRADEpro software (version 3.6) to present pooled effect estimates alongside corresponding certainty ratings in a structured and transparent format.

### Statistical analysis

All quantitative analyses were performed using RStudio (version 4.5.1) [19]. Effect sizes were synthesized using risk ratios (RRs) for binary outcomes and mean differences (MDs) for continuous variables, each reported with corresponding 95% confidence intervals (CIs). Forest plots were constructed to visually display pooled estimates. Between-study variability was accounted for using a random-effects modeling framework for all outcomes. This approach was selected to incorporate anticipated clinical and methodological diversity across studies and to provide effect estimates that reflect variability beyond sampling error. Fixed-effect assumptions were not applied. To evaluate the stability of pooled estimates, leave-one-out sensitivity analyses were conducted by sequentially omitting individual studies and reassessing both overall effect size and heterogeneity. Small-study effects and potential reporting bias were examined using Doi plots, accompanied by calculation of the Luis Furuya-Kanamori (LFK) index. LFK values between − 1 and + 1 were interpreted as indicating symmetry, values between ± 1 and ± 2 as minor asymmetry, and values exceeding ± 2 as major asymmetry. Statistical inconsistency across studies was quantified using the I^2^ statistic and the Cochrane Q test [20, 21]. I^2^ values were interpreted as reflecting low (< 25%), moderate (25–50%), high (50–75%), or substantial (> 75%) heterogeneity. A p-value below 0.05 for the Q statistic was considered indicative of statistically significant heterogeneity. To explore potential sources of heterogeneity, pre-specified subgroup analyses were conducted according to (1) graft type (studies exclusively reporting right lobe hepatectomy versus those including mixed graft types) and (2) study design (single-center studies versus multicenter registry analyses). These analyses were performed for outcomes demonstrating substantial heterogeneity (I^2^ > 75%) when a sufficient number of studies were available to allow meaningful subgroup comparisons.

Consistent with contemporary methodological recommendations, random-effects estimation was maintained irrespective of heterogeneity magnitude to enhance the generalizability of findings [22]. All analyses were stratified by population (donors versus recipients) to account for fundamental differences in operative procedures and complication mechanisms between these two groups. Formal tests for subgroup differences (interaction tests) were performed using the Q-test based on analysis of variance to determine whether effect estimates varied significantly between donors and recipients. Given the distinct clinical contexts, donor and recipient outcomes are presented separately throughout, and no pooled estimates combining both populations are reported.

## Results

### Study selection

**Fig. 1:**
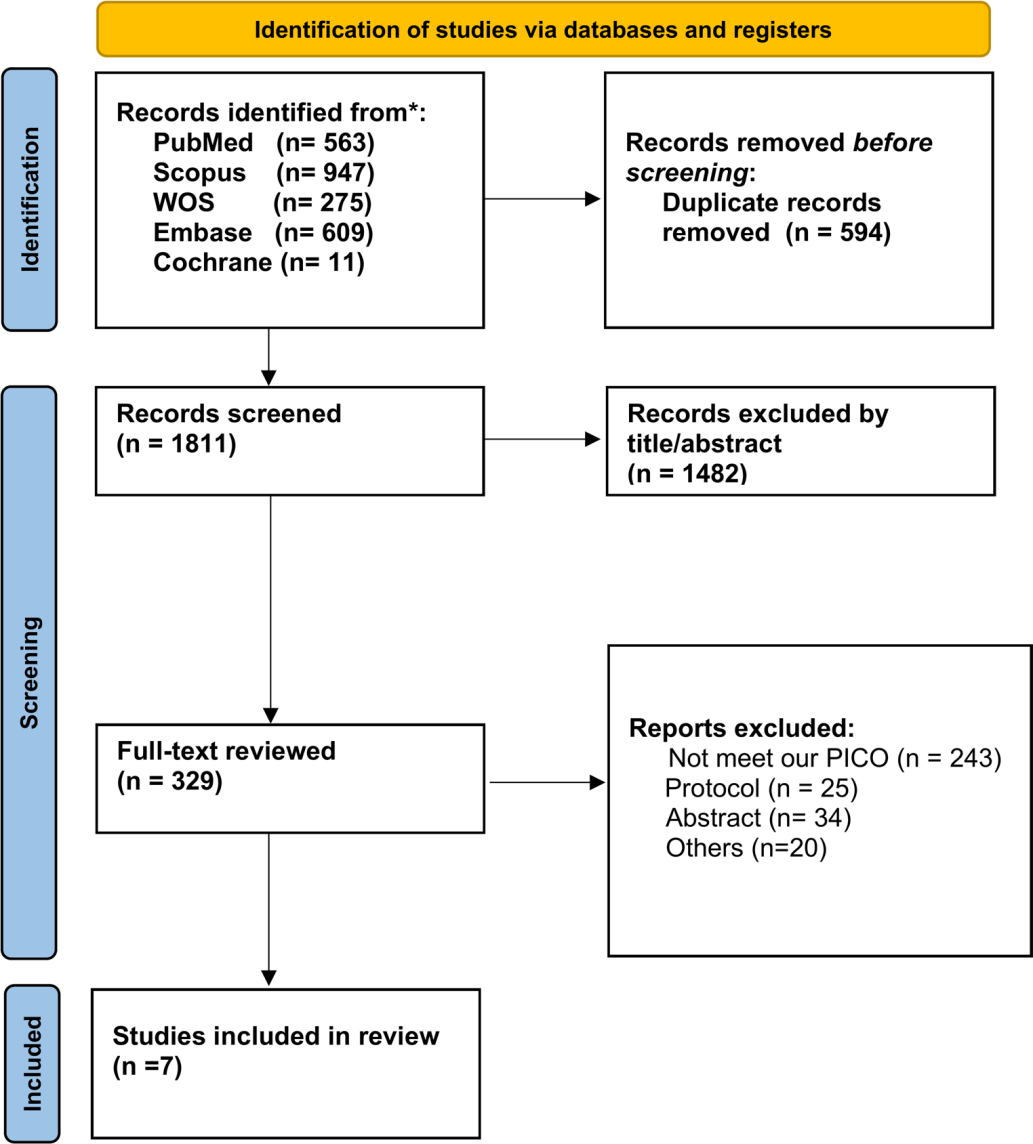
PRISMA 2020 flow diagram of study selection. Flow diagram summarizing records identified, screened, assessed for eligibility, and included in the qualitative synthesis and meta-analysis, with reasons for full-text exclusions

The search identified 2,405 records from the screened databases. Removal of 594 duplicate entries resulted in 1,811 unique citations for initial screening. Following title and abstract review, 1,482 records were excluded, and 329 articles proceeded to full-text assessment. Of these, 322 publications were excluded after detailed evaluation due to failure to meet predefined eligibility criteria, including non-comparative design, protocol-only publications, or availability of abstract data without complete reporting. Seven studies [10, 23–28] satisfied all inclusion criteria and were incorporated into both the qualitative synthesis and quantitative meta-analysis. A detailed overview of the screening pathway is presented in the PRISMA 2020 flow diagram **(**Fig. 1**)**.

### Demographics and study characteristics

Across the included studies, donor and recipient baseline profiles were largely comparable between robotic and laparoscopic groups **(**Table 1**)**. Donors were predominantly young adults, with reported mean ages generally between 29 and 38 years. Body mass index values were within the normal range (approximately 22–25 kg/m^2^), and the proportion of female donors was similar across surgical approaches. Recipient characteristics, when available, demonstrated comparable distributions between groups. Mean recipient age was typically in the fifth to sixth decade of life, with BMI values ranging from roughly 23 to 27 kg/m^2^ and no marked differences in sex distribution. Indicators of disease severity were also aligned, as reflected by overlapping MELD scores (commonly 11–16). Measures of graft adequacy, including graft-to-recipient weight ratio (GRWR), showed minimal variation between approaches, suggesting similar graft quality. Although certain large registry datasets did not provide detailed recipient-level variables, no meaningful baseline discrepancies were identified that would suggest substantial preoperative imbalance. The final analysis incorporated seven studies published between 2022 and 2025, representing more than 15,000 living donors **(**Table 2**)**.

**Table 1:** Baseline demographics of donors and recipients in studies comparing robotic and laparoscopic donor hepatectomy for liver transplantation

Study ID	Arms	Number in each group	Donor age (Years) Mean ± SD	Donor BMI (kg/m^2^) Mean ± SD	Donor gender (% Female)	Number in each group	Recipient age (Years) Mean ± SD	Recipient BMI (kg/m^2^) Mean ± SD	Recipient gender (% Female)	Recipient MELD Score Mean ± SD	GRWR Mean ± SD
Kim 2025 [10]	Robotic	71	31.7 ± 9.5	23.4 ± 2.3	33 (46.5%)	71	56.3 ± 9.0	24.4 ± 3.3	17 (23.9%)	12.4 ± 3.0	1.12 ± 0.23
	Laparoscopic	71	32.8 ± 9.9	23.2 ± 2.9	38 (53.5%)	71	55.6 ± 8.7	24.5 ± 3.9	16 (22.5%)	13.5 ± 6.8	1.14 ± 0.25
LDLT registry 2025 [26]	Laparoscopic	242	38.0 ± 11.1	25.0 ± 3.7	120 (49.6%)	NR	NR	NR	NR	NR	NR
	Robotic	632	30.0 ± 8.1	25.0 ± 3.7	202 (32.0%)	NR	NR	NR	NR	NR	NR
Haruki 2025 [23]	Laparoscopic	1,479	NR	NR	NR	NR	NR	NR	NR	NR	NR
	Robotic	236	NR	NR	NR	NR	NR	NR	NR	NR	NR
Raptis 2024 [27]	Laparoscopic	165	30 ± 6	24 ± 4	57 (35%)	129 (Peds) 36 (Adult)	Peds: 1.5 ± 2.2 Adult: 57 ± 14	Peds: 9.7 ± 5.2 Adult: 27 ± 6	Peds: 65 (50%) Adult: 16 (44%)	Adult: 22.2 ± 2.2	Peds: 2.2 ± 0.74 Adult: 0.85 ± 0.22
	Robotic	913	30 ± 7	25 ± 4	272 (30%)	341 (Peds) 572 (Adult)	Peds: 3.2 ± 5.2 Adult: 54 ± 14	Peds: 14.8 ± 10.4 Adult: 27 ± 6	Peds: 165 (48%) Adult: 222 (39%)	Adult: 20.4 ± 5.2	Peds: 1.9 ± 1.5 Adult: 0.85 ± 0.22
Troisi et al. 2023 [25]	Robotic	92	30.0 ± 9.1	22.9 ± 2.3	44 (47.8%)	50	55.4 ± 10.5	23.5 ± 3.8	15 (30.0%)	14.0 ± 6.9	1.1 ± 0.2
	Laparoscopic	92	29.8 ± 9.3	23.3 ± 3.2	47 (51.1%)	50	55.1 ± 9.2	23.3 ± 3.2	15 (30.0%)	14.6 ± 6.7	1.1 ± 0.3
Kim 2022 [24]	Robotic	102	30.7 ± 9.4	22.8 ± 2.5	55 (53.9%)	102	56.5 ± 8.4	24.0 ± 3.4	22 (21.6%)	12.1 ± 5.0	1.1 ± 0.2
	Laparoscopic	69	30.4 ± 10.6	22.7 ± 2.7	36 (52.2%)	69	52.7 ± 9.3	24.7 ± 4.1	21 (30.4%)	16.5 ± 7.6	1.1 ± 0.3
Rho et al. 2022 [28]	Robotic	52	28.6 ± 8.7	22.4 ± 2.1	26 (50.0%)	52	56.5 ± 6.6	24.3 ± 3.3	11 (21.2%)	11.5 ± 5.1	1.1 ± 0.2
	Laparoscopic	118	36.9 ± 12.1	23.3 ± 2.5	44 (37.3%)	118	53.8 ± 11.2	23.8 ± 3.6	38 (32.2%)	16.2 ± 8.3	1.2 ± 0.3

BMI: Body Mass Index, MELD: Model for End-Stage Liver Disease, GRWR: Graft-to-Recipient Weight Ratio, NR: Not Reported, Peds: Pediatric, SD: Standard Deviation, kg/m^2^: Kilograms per Square Meter

**Table 2:** This table outlines the characteristics of the included studies and their respective operative protocols

Study ID	Study design	Country	Total sample size	Center volume	Robotic platform	Graft type	Key indications for transplantation	Duration of follow-up
Kim et al., 2025 [10]	Single-center retrospective	South Korea	235 donors (RDRH: 117, LDRH: 118)	High	Da vinci surgical system	Right hepatectomy	Hepatocellular carcinoma and ESLD	NR
LDLT registry, 2025 [26]	Multicenter prospective	Global (62 centers, 27 countries)	2,600 donors	Mixed (predominantly high)	NR	Right, left, left lateral	NR	Up to 90 days post-op
Haruki et al., 2025 [23]	Multicenter retrospective	Japan, India, Korea, Turkey, Brazil, etc	10,025 donors	Mixed (predominantly high)	NR	Right, left, left lateral	NR	NR
Raptis et al., 2024 [27]	Single-center prospective registry	Saudi Arabia	1,724 donor-recipient pairs (3,448 total)	High	Da vinci Xi	Right, left, left lateral	Pediatric: metabolic, biliary, ALF; adult: viral hepatitis, NASH, HCC, ALD, autoimmune hepatitis, etc	In-hospital and up to 5-year survival tracking
Troisi et al., 2023 [25]	Multicenter retrospective	South Korea, USA, Taiwan, Italy	1,194 donor hepatectomies	High	NR	Right hepatectomy	Alcohol-associated cirrhosis, viral (HBV/HCV) cirrhosis, HCC, NASH, autoimmune hepatitis, cholestatic, cryptogenic, acute liver failure, metabolic	Mean follow-up 18 months (recipients)
Kim et al., 2022 [24]	Single-center retrospective	South Korea	171 donors (RUDR: 102, LLDRH: 69)	High	Da vinci surgical system	Right hepatectomy	Hepatocellular carcinoma and ESLD	NR
Rho et al., 2022 [28]	Single-center retrospective	South Korea	52 donors (RUDR)	High	Da vinci surgical system	Right hepatectomy	Hepatocellular carcinoma and ESLD	12 months

It details the study design, country of origin, sample size, robotic platform used, type of graft, indications for transplantation, and duration of follow-up for studies comparing robotic and laparoscopic living donor hepatectomy

**LDRH:** Laparoscopic Donor Right Hepatectomy, **ESLD:** End-Stage Liver Disease, **LDLT:** Living Donor Liver Transplantation, **NR:** Not Reported, **ALF:** Acute Liver Failure, **NASH:** Non-Alcoholic Steatohepatitis, **HCC:** Hepatocellular Carcinoma, **ALD:** Alcoholic Liver Disease, **HBV/HCV:** Hepatitis B Virus / Hepatitis C Virus, **RUDR:** Robotic Living Donor Right Hepatectomy

To address potential heterogeneity arising from the inclusion of studies reporting mixed graft types (LDLT registry 2025; Haruki et al. 2025; Raptis et al. 2024), sensitivity analyses restricted to studies exclusively reporting right lobe hepatectomy (Kim et al. 2025; Troisi et al. 2023; Kim et al. 2022; Rho et al. 2022) were conducted for key outcomes where sufficient data were available. These analyses generally confirmed the direction and magnitude of the primary pooled estimates, although statistical power was reduced because of the smaller number of eligible studies. Detailed results are not shown but are available upon request.

Most investigations were retrospective in design and originated primarily from high-volume centers in East Asia, with additional contributions from multinational collaborations. Robotic procedures were predominantly performed using the da Vinci platform, and right lobe grafts constituted the majority of cases. Indications for transplantation were heterogeneous, and follow-up reporting varied considerably, ranging from early postoperative outcomes to longitudinal data extending up to five years.

### Risk of bias

**Fig. 2:**
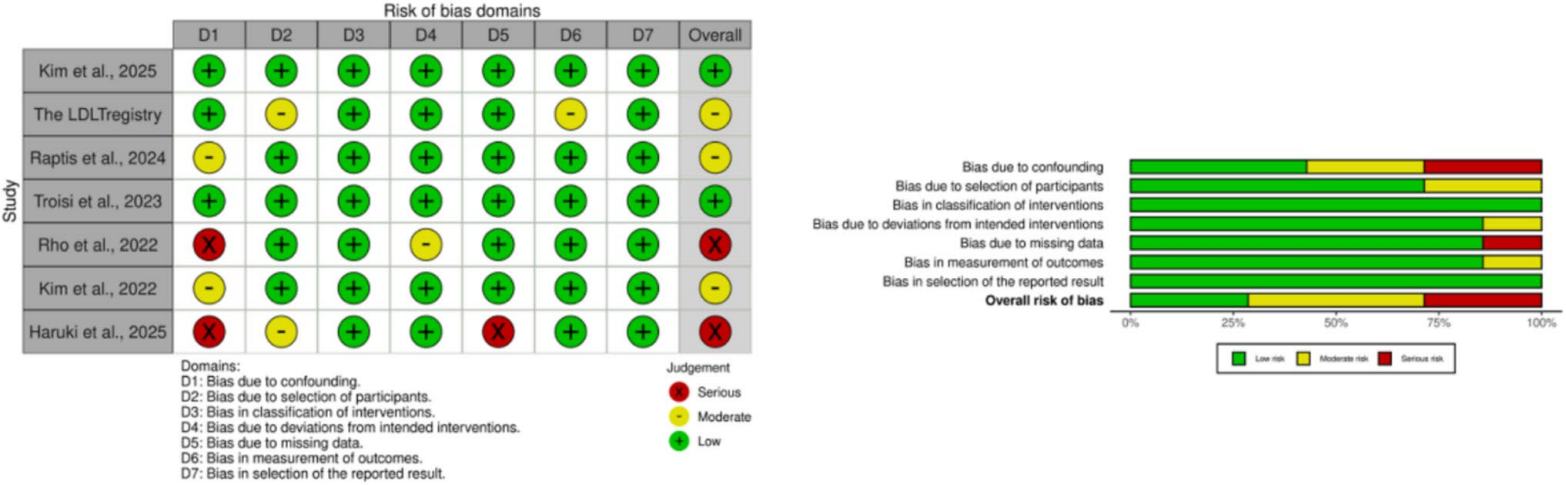
Risk-of-bias assessment (ROBINS-I). Risk-of-bias evaluation for included non-randomized comparative studies using the ROBINS-I tool. Visualizations (traffic-light and summary plots) were generated using the robvis web application

Evaluation using the ROBINS-I framework **(**Fig. 2**)** demonstrated generally acceptable methodological quality among the included studies. Most domains—particularly classification of interventions, adherence to intended interventions, completeness of outcome data, outcome assessment, and reporting practices—were judged to carry a low risk of bias. In contrast, concerns were more frequently noted in domains related to confounding and participant selection. These factors contributed to moderate risk assessments in several studies and serious risk ratings in a minority. Taken together, the overall methodological profile of the included evidence can be characterized as predominantly low to moderate risk. While the pooled findings appear methodologically supported, the possibility of residual confounding remains the principal limitation inherent to the observational nature of the data.

### Certainty of evidence (GRADE)

Overall, the strength of evidence across evaluated outcomes was generally rated as low to very low. While robotic living donor hepatectomy was associated with reduced transfusion requirements and favorable recipient morbidity outcomes, these estimates must be considered in light of methodological limitations. Residual confounding, inter-study variability, and indications of reporting bias reduce confidence in the magnitude and stability of the observed effects. Accordingly, high-quality randomized controlled trials are necessary to establish more definitive conclusions **(ESM 3)**.

## Outcomes of recipients and donors

### Outcomes of donors

#### Operative time

**Fig. 3:**
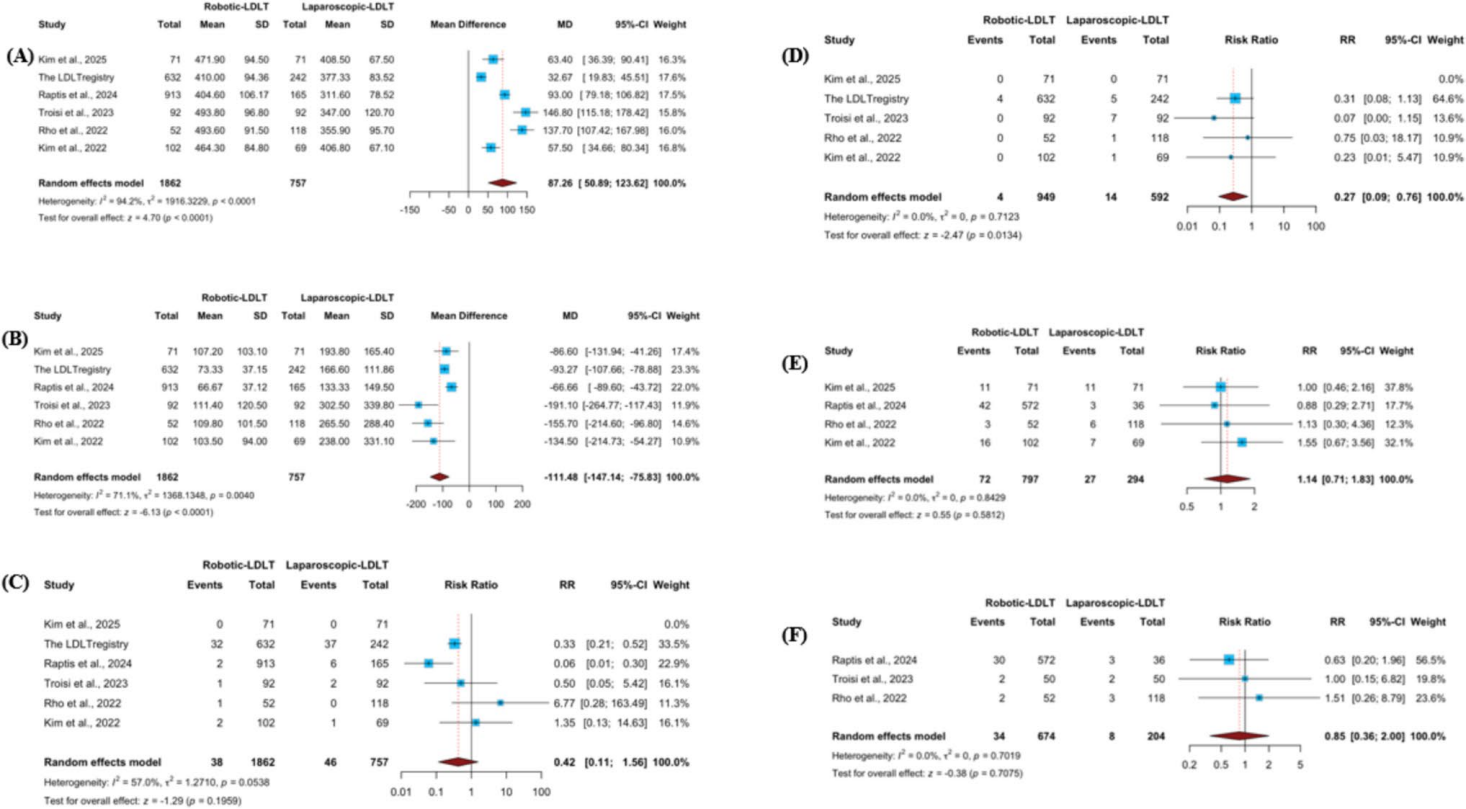
Donor operative outcomes and recipient vascular outcomes comparing robotic and laparoscopic living donor hepatectomy. Forest plots comparing robotic living donor hepatectomy (R-LDH) and laparoscopic living donor hepatectomy (L-LDH) for the following outcomes: (**A**) operative time, (**B**) estimated blood loss, (**C**) conversion to open surgery, (**D**) perioperative blood transfusion, (**E**) recipient overall vascular complications, and (**F**) recipient hepatic artery thrombosis. Continuous variables are presented as mean differences, and dichotomous variables as risk ratios, each with corresponding 95% confidence intervals calculated using random-effects models

Operative duration was reported in six studies. Meta-analytic synthesis revealed that robotic living donor hepatectomy was associated with a significantly longer operative time compared with the laparoscopic approach (mean difference [MD] 87.26 min; 95% CI 50.89–123.62; p < 0.0001) **(**Fig. 3A**)**. Considerable between-study variability was observed (I^2^ = 94.2%, p < 0.0001). Robustness testing through sequential exclusion of individual studies did not materially alter the direction or statistical significance of the effect estimate, and heterogeneity remained substantial **(ESM2 Fig. S2.1A)**. Assessment of small-study effects using the Doi plot demonstrated marked asymmetry, with an LFK index of 2.66, suggesting the presence of significant publication bias **(ESM2 Fig. S2.1B)**. Subgroup analyses by graft type and study design showed that robotic hepatectomy was associated with longer operative time in both right-lobe–only (MD 100.53 min; 95% CI 54.04–147.02) and mixed-graft studies (MD 62.78 min; 95% CI 3.66–121.90), without a significant subgroup difference (p = 0.325) (ESM2 Fig. S2.11). A similar pattern was observed across study designs, with longer operative time in single-center studies (MD 87.28 min; 95% CI 52.89–121.67) but not in multicenter registries (MD 88.78 min; 95% CI − 23.05–200.61), and no significant interaction (p = 0.979) (ESM2 Fig. S2.12).

#### Estimated blood loss

Data on intraoperative blood loss was available from six studies. Quantitative pooling indicated that robotic donor hepatectomy was associated with significantly lower estimated blood loss compared with the laparoscopic technique (MD − 111.48 mL; 95% CI − 147.14 to − 75.83; p < 0.0001) **(**Fig. 3B**)**. Moderate-to-high heterogeneity was detected across studies (I^2^ = 71.1%, p = 0.0040). Sequential omission of individual studies during sensitivity analysis did not materially change the magnitude or statistical significance of the observed effect, and heterogeneity persisted **(ESM2 Fig. S2.2A)**. Evaluation of reporting bias using the Doi plot demonstrated pronounced asymmetry, with an LFK index of − 3.6, consistent with substantial small-study effects **(ESM2 Fig. S2.2B)**.

#### Conversion to open hepatectomy

Conversion to an open procedure was evaluated in six studies. When pooled, the comparison between robotic and laparoscopic donor hepatectomy did not demonstrate a statistically significant difference in conversion rates (RR 0.42; 95% CI 0.11–1.56; p = 0.1959) **(**Fig. 3C**)**. Between-study variability was moderate (I^2^ = 57%, p = 0.0538). Influence analysis revealed that removal of the study by Rho et al. (2022) altered the pooled estimate, yielding a significantly lower likelihood of conversion in the robotic group (RR 0.29; 95% CI 0.10–0.83; p = 0.0216), with reduced heterogeneity (I^2^ = 48.1%) **(ESM2 Fig. S2.3A)**. Assessment of potential reporting bias demonstrated marked asymmetry on the Doi plot, reflected by an LFK index of 3.36, consistent with substantial small-study effects **(ESM2 Fig. S2.3B)**.

#### Blood transfusion

Transfusion requirements were reported in five studies. Aggregated analysis indicated a significantly lower likelihood of perioperative blood transfusion among donors undergoing robotic hepatectomy compared with the laparoscopic approach (RR 0.27; 95% CI 0.09–0.76; p = 0.0134) **(**Fig. 3D**)**. No statistical heterogeneity was detected across studies (I^2^ = 0%, p = 0.7123). Sequential exclusion of individual studies during sensitivity testing did not materially influence the pooled estimate, and heterogeneity remained negligible **(ESM2 Fig. S2.4A)**. Inspection of the Doi plot demonstrated mild asymmetry, with an LFK index of 2.66, raising the possibility of small-study effects **(ESM2 Fig. S2.4B)**.

### Outcomes of the recipient

In all included studies, recipient hepatectomy and graft implantation were uniformly performed using a conventional open surgical approach. Accordingly, the following analyses reflect recipient clinical outcomes and are not confounded by variability in recipient operative technique; any observed differences are attributable to the donor surgical approach rather than recipient implantation methods.

#### Overall vascular complications

Four studies provided comparative data on recipient vascular complications. Combined estimates did not demonstrate a statistically meaningful difference between robotic and laparoscopic donor hepatectomy (RR 1.14; 95% CI 0.71–1.83; p = 0.5812) **(**Fig. 3E**)**. No evidence of between-study heterogeneity was identified (I^2^ = 0%, p = 0.5812). Robustness analysis confirmed the stability of these findings, as sequential removal of individual studies did not materially alter the effect size or heterogeneity metrics **(ESM2 Fig. S2.5A)**. Evaluation of potential small-study effects showed near symmetry on the Doi plot, with an LFK index of − 0.44, suggesting no relevant publication bias **(ESM2 Fig. S2.5B)**.

#### Hepatic Artery Thrombosis (HAT)

Incidence of hepatic artery thrombosis was examined in three studies. Meta-analytic pooling did not reveal a statistically significant difference between robotic and laparoscopic donor hepatectomy (RR 0.85; 95% CI 0.36–2.00; p = 0.7075) **(**Fig. 3F**)**. No measurable heterogeneity was detected across studies (I^2^ = 0%, p = 0.7019). Sensitivity testing confirmed the consistency of the findings, as sequential omission of individual studies did not meaningfully affect the overall estimate or heterogeneity measures **(ESM2 Fig. S2.5C)**. Assessment of reporting bias showed minimal asymmetry on the Doi plot, with an LFK index of 0.77, indicating no substantial evidence of small-study effects **(ESM2 Fig. S2.5D)**.

#### Reoperation rate

**Fig. 4:**
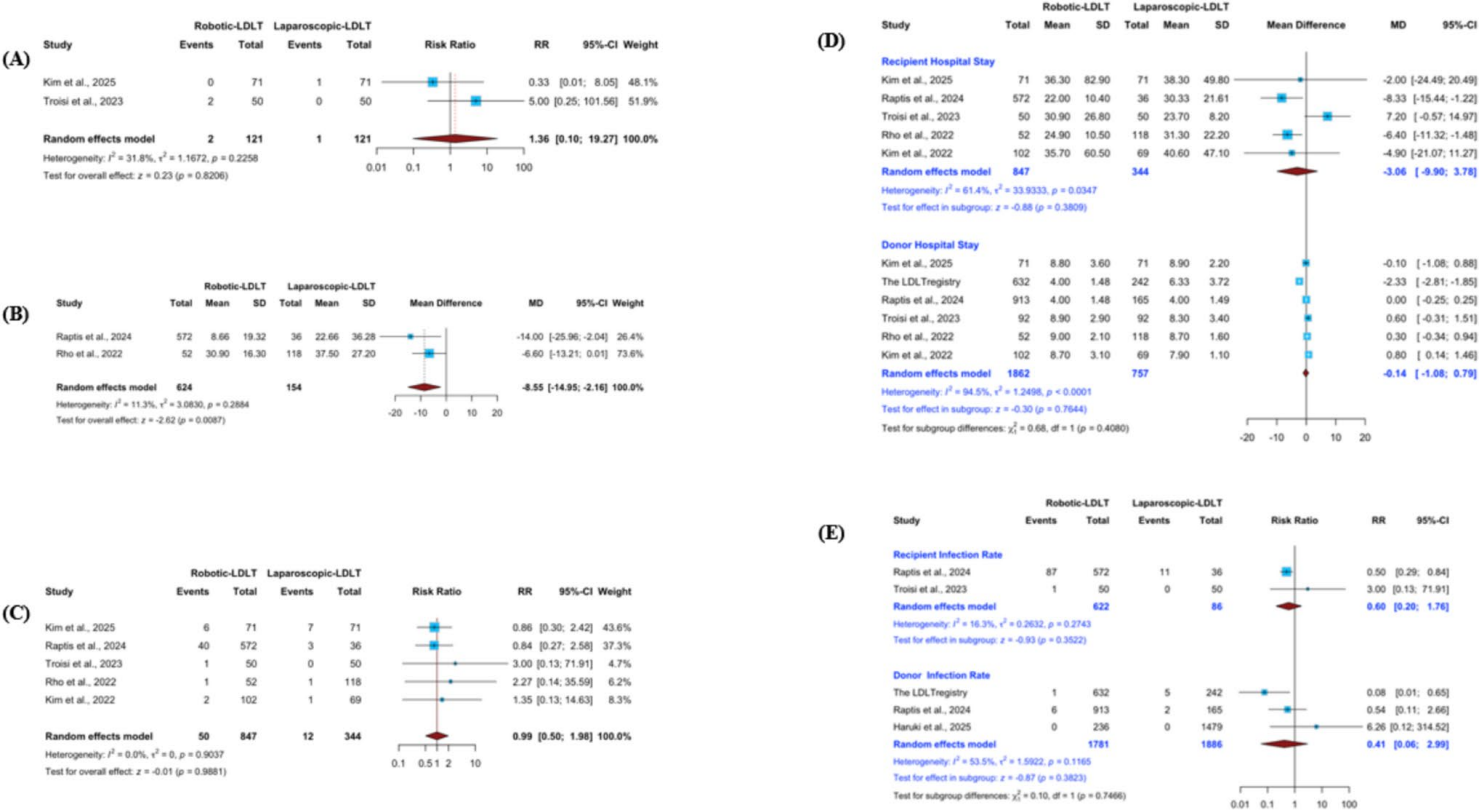
Perioperative outcomes in donors and recipients following robotic versus laparoscopic living donor hepatectomy. Forest plots comparing robotic (R-LDH) and laparoscopic (L-LDH) donor hepatectomy. Donor outcomes are presented in the upper panels: (**A**) reoperation rate, (**B**) Comprehensive Complication Index (CCI), and (**C**) mortality. Recipient outcomes are presented separately in the lower panels: (**D**) length of hospital stay and (**E**) infection rate. Effect estimates are shown with 95% confidence intervals derived from random-effects models. Donor and recipient outcomes were analyzed independently and should not be interpreted as combined estimates

Comparative data on reintervention were available from two studies. Pooled effect estimates did not demonstrate a statistically significant difference in reoperation rates between robotic and laparoscopic donor hepatectomy (RR 1.36; 95% CI 0.10–19.27; p = 0.8206) **(**Fig. 4A**)**. Between-study variability was moderate (I^2^ = 31.8%, p = 0.2258).

#### Comprehensive Complication Index (CCI)

Two studies provided data on the Comprehensive Complication Index (CCI). Quantitative synthesis indicated that donors undergoing robotic hepatectomy experienced a significantly lower cumulative complication burden compared with those treated laparoscopically (MD − 8.55; 95% CI − 14.95 to − 2.16; p = 0.0087) **(**Fig. 4B**)**. Statistical heterogeneity was minimal across studies (I^2^ = 11.3%, p = 0.2884).

#### Mortality rate

Mortality outcomes were available from five studies. When effect estimates were combined, no statistically significant difference was observed between robotic and laparoscopic donor hepatectomy (RR 0.99; 95% CI 0.50–1.98; p = 0.9881) **(**Fig. 4C**)**. No evidence of inter-study heterogeneity was detected (I^2^ = 0%, p = 0.9037). Influence analysis confirmed the stability of the pooled result, as sequential exclusion of individual studies did not meaningfully alter the effect estimate or heterogeneity measures **(ESM2 Fig. S2.6A)**. However, evaluation of potential small-study effects revealed marked asymmetry on the Doi plot, with an LFK index of 4.89, suggesting the presence of substantial reporting bias **(ESM2 Fig. S2.6B)**.

### Shared donor and recipient outcomes

#### Length of hospital stay—donors

Six studies provided data on donor hospital stay. Pooled analysis demonstrated no statistically significant difference between robotic and laparoscopic donor hepatectomy (MD − 0.14 days; 95% CI − 1.08 to 0.79; p = 0.7644) (Fig. 4D). Substantial heterogeneity was detected (I^2^ = 94.5%, p < 0.0001). Exploratory sensitivity analysis excluding the LDLT registry reduced heterogeneity to a moderate level (I^2^ = 39.2%), although the pooled estimate remained statistically nonsignificant (ESM2 Fig. S2.7C). Assessment of small-study effects demonstrated near symmetry on the Doi plot (LFK index 0.33), suggesting no meaningful publication bias (ESM2 Fig. S2.7D). Subgroup analyses for donor hospital stay showed a slightly longer stay in right-lobe–only studies (MD 0.46 days; 95% CI 0.08–0.84) but no difference in mixed-graft studies (MD − 1.16 days; 95% CI − 3.44–1.13), with no significant subgroup interaction (p = 0.172) (ESM2 Fig. S2.13). Stratification by study design likewise showed no significant differences in single-center (MD 0.22 days; 95% CI − 0.16–0.61) or multicenter studies (MD − 0.89 days; 95% CI − 3.76–1.98), without a significant subgroup interaction (p = 0.450) (ESM2 Fig. S2.14).

#### Length of hospital stay—recipients

Data on recipient length of hospital stay were available from five studies. Pooled analysis demonstrated no statistically significant difference between robotic and laparoscopic donor hepatectomy in recipient hospitalization duration (MD − 3.06 days; 95% CI − 9.90 to 3.78; p = 0.3809) (Fig. 4D). Moderate heterogeneity was observed across studies (I^2^ = 61.4%, p = 0.0347). Sensitivity analysis identified the study by Troisi et al. (2023) as a major contributor to heterogeneity. Removal of this study reduced heterogeneity to 0% and resulted in a significantly shorter hospital stay in the robotic group (MD − 6.75 days; 95% CI − 10.62 to − 2.89; p = 0.0006) (ESM2 Fig. S2.7A). Assessment of small-study effects demonstrated pronounced asymmetry (LFK index 2.18), suggesting potential reporting bias (ESM2 Fig. S2.7B). For recipient hospital stay, subgroup analysis by study design showed a significantly shorter hospital stay in single-center studies (MD − 6.75 days; 95% CI − 10.62 to − 2.89), whereas the multicenter study showed no statistically significant difference (MD 7.20 days; 95% CI − 0.57 to 14.97) (ESM2 Fig. S2.15). A significant subgroup interaction was observed (p = 0.0016), suggesting potential variability across study designs.

#### Infections – donors

Three studies provided data on donor postoperative infections. Pooled analysis demonstrated no statistically significant difference between robotic and laparoscopic donor hepatectomy (RR 0.41; 95% CI 0.06–2.99; p = 0.3823) (Fig. 4E). Moderate heterogeneity was detected (I^2^ = 53.5%, p = 0.1165). Sensitivity analysis excluding the LDLT registry reduced heterogeneity to a low level (I^2^ = 22.2%) while preserving the direction and statistical interpretation of the pooled estimate (ESM2 Fig. S2.9G). Assessment of small-study effects demonstrated mild asymmetry on the Doi plot (LFK index 1.04), suggesting the possibility of small-study effects (ESM2 Fig. S2.9H).

#### Infections – recipients

Two studies provided data on recipient postoperative infections. Pooled analysis demonstrated no statistically significant difference between grafts procured robotically and laparoscopically (RR 0.60; 95% CI 0.20–1.76; p = 0.3522) (Fig. 4E). Minimal heterogeneity was observed (I^2^ = 16.3%, p = 0.2743).

#### Overall biliary complications – donors

**Fig. 5:**
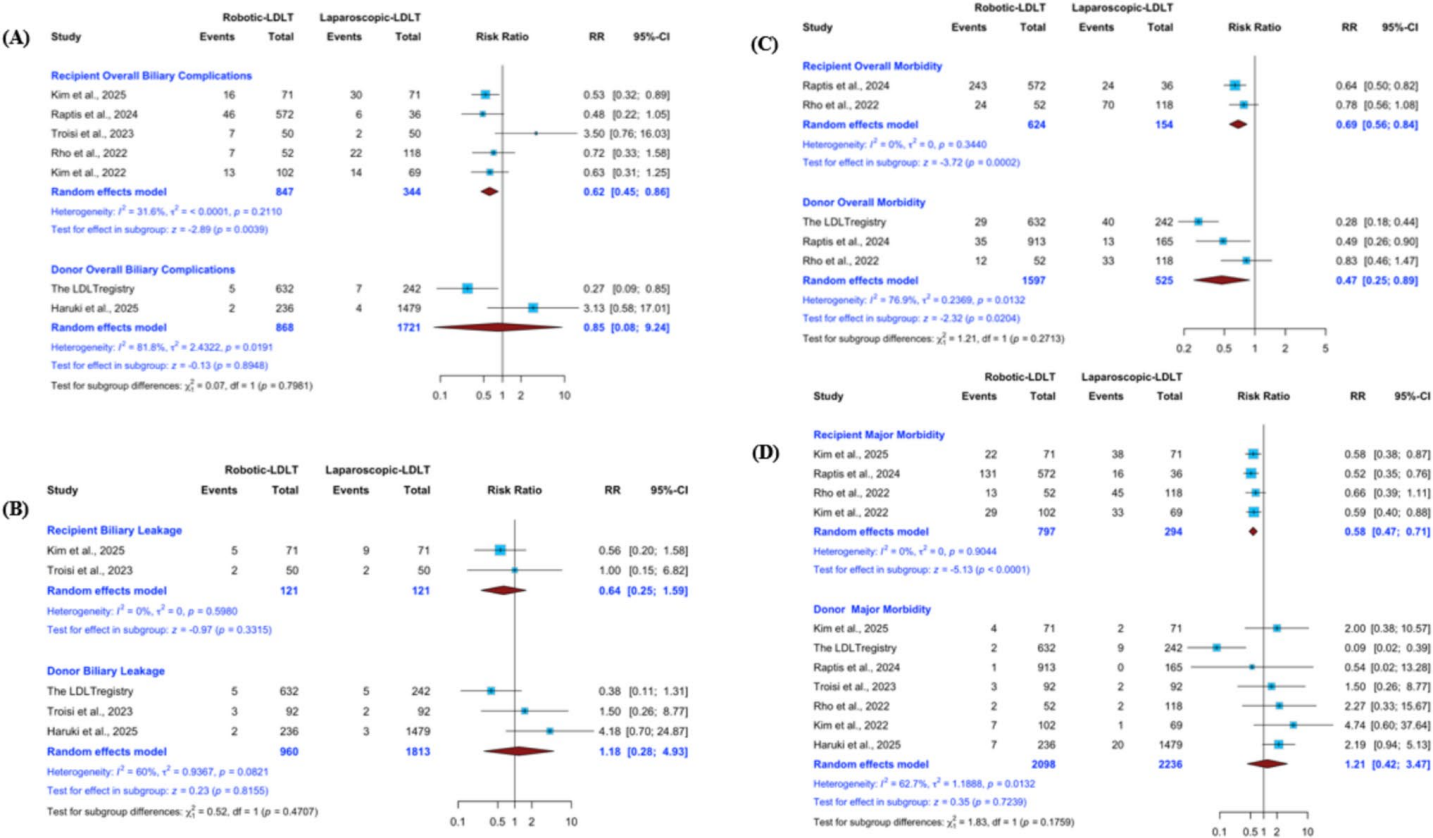
Biliary complications and postoperative morbidity in donors and recipients following robotic versus laparoscopic living donor hepatectomy. Forest plots comparing robotic (R-LDH) and laparoscopic (L-LDH) donor hepatectomy for: (**A**) overall biliary complications, (**B**) biliary leakage, (**C**) overall morbidity, and (**D**) major morbidity (Clavien–Dindo grade ≥ III). Donor and recipient outcomes are presented separately within each analysis and were evaluated independently. Pooled risk ratios with corresponding 95% confidence intervals were calculated using random-effects models

Two studies provided data on donor overall biliary complications. Pooled analysis demonstrated no statistically significant difference between robotic and laparoscopic donor hepatectomy (RR 0.85; 95% CI 0.08–9.24; p = 0.8948) (Fig. 5A). Substantial heterogeneity was detected (I^2^ = 81.8%, p = 0.0191).

#### Overall biliary complications – recipients

Five studies provided data on recipient overall biliary complications. Pooled analysis demonstrated a significantly lower incidence of biliary complications in recipients of robotically procured grafts compared with laparoscopically procured grafts (RR 0.62; 95% CI 0.45–0.86; p = 0.0039) (Fig. 5A). Moderate heterogeneity was observed (I^2^ = 31.6%, p = 0.2110). Sensitivity analysis showed that removal of the study by Troisi et al. (2023) eliminated heterogeneity (I^2^ = 0%) while preserving the direction and statistical significance of the pooled estimate (RR 0.57; 95% CI 0.41–0.80; p = 0.0009) (ESM2 Fig. S2.8A). Assessment of small-study effects demonstrated marked asymmetry on the Doi plot (LFK index 2.86), suggesting potential reporting bias (ESM2 Fig. S2.8B).

#### Biliary leakage – donors

Three studies provided data on donor biliary leakage. Pooled analysis demonstrated no statistically significant difference between robotic and laparoscopic donor hepatectomy (RR 1.18; 95% CI 0.28–4.93; p = 0.8155) (Fig. 5B). Moderate heterogeneity was detected (I^2^ = 60%, p = 0.0821). Sensitivity analysis excluding the LDLT registry eliminated heterogeneity (I^2^ = 0%) without altering the overall interpretation of the pooled estimate (ESM2 Fig. S2.8C). Assessment of small-study effects demonstrated near symmetry on the Doi plot (LFK index 0.48), indicating no substantial evidence of publication bias (ESM2 Fig. S2.8D).

#### Biliary leakage – recipients

Two studies provided data on recipient biliary leakage. Pooled analysis demonstrated no statistically significant difference between grafts procured robotically and laparoscopically (RR 0.64; 95% CI 0.25–1.59; p = 0.3315) (Fig. 5B). No heterogeneity was observed (I^2^ = 0%, p = 0.5980).

#### Overall morbidity – donors

Three studies provided data on donor overall morbidity. Pooled analysis demonstrated a significantly lower risk of overall complications following robotic donor hepatectomy compared with the laparoscopic approach (RR 0.47; 95% CI 0.25–0.89; p = 0.0204) (Fig. 5C). Substantial heterogeneity was detected (I^2^ = 76.9%, p = 0.0132). Sensitivity analysis excluding the study by Rho et al. (2022) reduced heterogeneity to a moderate level (I^2^ = 51.7%) while preserving the direction and statistical significance of the pooled estimate (ESM2 Fig. S2.9A). Assessment of small-study effects demonstrated no asymmetry on the Doi plot (LFK index 0.4), suggesting no meaningful publication bias (ESM2 Fig. S2.9B).

#### Overall morbidity – recipients

Two studies provided data on recipient overall morbidity. Pooled analysis demonstrated a significantly lower risk of overall complications in recipients of robotically procured grafts compared with laparoscopically procured grafts (RR 0.69; 95% CI 0.56–0.84; p = 0.0002) (Fig. 5C). No heterogeneity was observed (I^2^ = 0%, p = 0.3440).

#### Major morbidity – donors

Seven studies reported donor major complications (Clavien–Dindo grade ≥ IIIa). Initial pooled analysis demonstrated no statistically significant difference between robotic and laparoscopic approaches (RR 1.21; 95% CI 0.42–3.47; p = 0.7239), with substantial heterogeneity across studies (I^2^ = 62.7%, p = 0.0132) (Fig. 5D). Sensitivity analysis revealed that exclusion of the LDLT registry eliminated heterogeneity (I^2^ = 0%) and altered the direction of the pooled estimate, suggesting a higher risk of major morbidity in the robotic group (RR 2.11; 95% CI 1.14–3.89; p = 0.0169) (ESM2 Fig. S2.9E). This finding indicates that the large registry dataset had a substantial influence on the pooled estimate. Minor asymmetry was observed on the Doi plot (LFK index − 1.41), suggesting possible publication bias (ESM2 Fig. S2.9F).

#### Major morbidity – recipients

Four studies provided data on recipient major morbidity, defined as Clavien–Dindo grade ≥ IIIa. Pooled analysis demonstrated a significantly lower incidence of major complications in recipients of robotically procured grafts compared with laparoscopically procured grafts (RR 0.58; 95% CI 0.47–0.71; p < 0.0001) (Fig. 5D). No heterogeneity was observed (I^2^ = 0%, p = 0.9044). Sensitivity analysis demonstrated that sequential exclusion of individual studies did not materially alter the magnitude, direction, or heterogeneity of the pooled estimate (ESM2 Fig. S2.9C). Assessment of small-study effects demonstrated symmetric distribution on the Doi plot (LFK index 0.61), supporting the stability of the pooled estimate (ESM2 Fig. S2.9D).

### Outcomes related to time

#### Warm and cold ischemia time

**Fig. 6:**
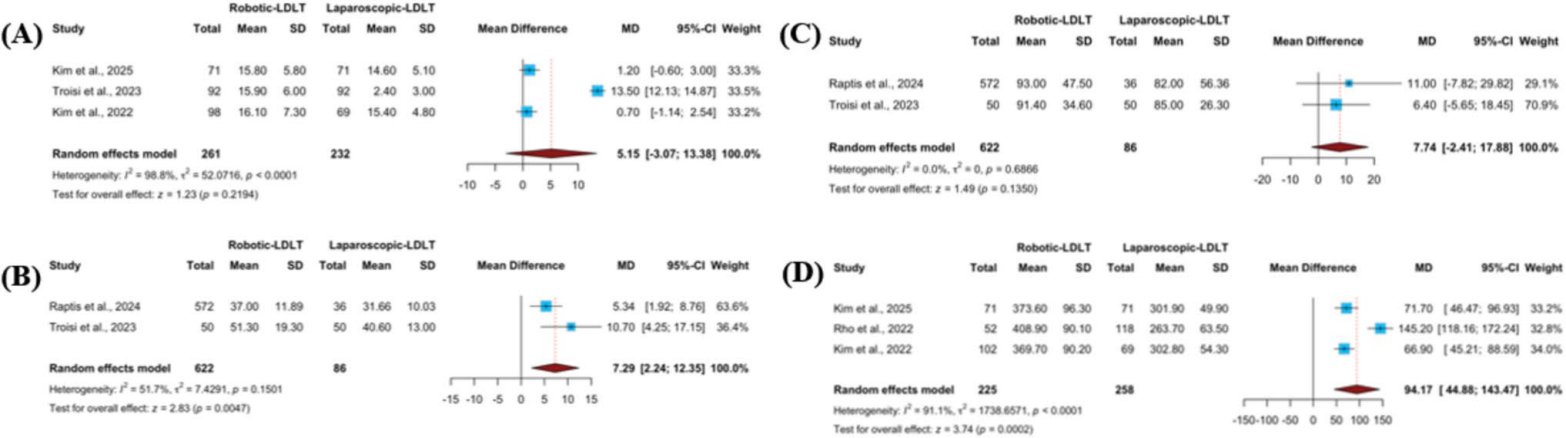
Ischemia-related and graft extraction timing outcomes comparing robotic and laparoscopic living donor hepatectomy. Forest plots comparing R-LDH and L-LDH for: (**A**) first warm ischemia time, (**B**) second warm ischemia time, (**C**) cold ischemia time, and (**D**) graft-out time. Outcomes are presented as mean differences with corresponding 95% confidence intervals derived using random-effects meta-analytic models

Data on first warm ischemia time were available from three studies, while second warm ischemia time was reported in two. Pooled analysis did not identify a statistically significant difference in first warm ischemia duration between robotic and laparoscopic donor hepatectomy (MD 5.15 min; 95% CI − 3.07 to 13.38; p = 0.2194) **(**Fig. 6A**)**. Considerable heterogeneity was present (I^2^ = 98.8%, p < 0.0001). Sensitivity exploration indicated that exclusion of Troisi et al. (2023) eliminated statistical heterogeneity (I^2^ = 0%); however, the effect estimate remained nonsignificant **(ESM2 Fig. S2.10A)**. Examination of the Doi plot demonstrated near symmetry (LFK index 0.86), suggesting no meaningful evidence of reporting bias **(ESM2 Fig. S2.10B)**. In contrast, second warm ischemia time was significantly prolonged in the robotic group (MD 7.29 min; 95% CI 2.24–12.35; p = 0.0047), with moderate between-study variability (I^2^ = 51.7%, p = 0.1501) **(**Fig. 6B**)**. Analysis of cold ischemia time showed no statistically significant difference between techniques (MD 7.74 min; 95% CI − 2.41 to 17.88; p = 0.1350), and no heterogeneity was detected (I^2^ = 0%, p = 0.6866) **(**Fig. 6C**)**.

#### Graft-out time

Three studies evaluated graft-out time. Combined estimates demonstrated a significantly longer extraction time in the robotic group compared with the laparoscopic approach (MD 94.17 min; 95% CI 44.88–143.47; p = 0.0002) **(**Fig. 6D**)**. Considerable heterogeneity was observed across studies (I^2^ = 91.1%, p < 0.0001). Because only a limited number of studies reported this outcome, subgroup analyses according to graft type or study design were not feasible. Influence analysis showed that removal of the study by Rho et al. (2022) eliminated heterogeneity (I^2^ = 0%) while maintaining the statistical significance of the association **(ESM2 Fig. S2.10C)**. Inspection of the Doi plot indicated mild asymmetry (LFK index 1.79), suggesting the presence of potential small-study effects **(ESM2 Fig. S2.10D)**.

## Discussion

This analysis synthesizing data from seven comparative studies and more than 15,000 living donors suggests that robotic living donor hepatectomy offers measurable perioperative advantages over the laparoscopic approach in appropriately experienced centers. While robotic procedures were associated with longer operative duration, they were accompanied by reduced intraoperative blood loss, lower transfusion rates, and decreased overall donor morbidity. Importantly, recipients of grafts retrieved using robotic techniques also demonstrated favorable outcomes, including lower rates of overall and major complications and fewer biliary events. In contrast, vascular complication rates and mortality appeared comparable between the two minimally invasive strategies.

An important methodological consideration in this analysis is the strict separation of donor and recipient outcomes throughout. Although both populations are intrinsically linked through the transplantation procedure, the operative interventions differ fundamentally: donors undergo pure hepatectomy, whereas recipients undergo total hepatectomy followed by graft implantation. Accordingly, the mechanisms underlying postoperative complications, the risk profiles, and the clinical implications are distinct between these groups. All analyses were therefore stratified by population, and no combined estimates are presented. Formal interaction tests confirmed that effect sizes did not differ significantly between donors and recipients for most outcomes (p > 0.05 for all interaction tests), supporting the validity of examining these populations separately within the same analytical framework.

These findings are consistent with contemporary evidence suggesting superior outcomes with robotic living donor hepatectomy. In a recent multicenter prospective registry, 646 (37%) donors underwent open hepatectomy, 165 (10%) laparoscopic, and 913 (53%) robotic procedures. The robotic group exhibited the lowest overall donor morbidity compared with laparoscopic and open approaches (4%, 8%, and 16%, respectively; P < 0.001). Similarly, major morbidity among pediatric and adult recipients following robotic hepatectomy was 15% and 23%, compared with 25% and 44% for laparoscopic surgery and 19% and 31% for open surgery, respectively (P = 0.033 for pediatric recipients; P < 0.001 for adults) [27].

A recent meta-analysis of propensity score–matched studies concluded that the robotic approach offers significant advantages with respect to estimated blood loss and open conversion rates [29]. Additionally, a retrospective analysis focusing on left hemihepatectomy demonstrated reduced intraoperative blood loss with the robotic approach compared with laparoscopy, despite no significant difference in operative duration [30]. Conversely, Aboudou et al. reported no significant differences in blood loss, hospital stay, or conversion rates, although robotic hepatectomy was associated with longer operative time [31]. In our analysis, the observed reduction in estimated blood loss with R-LDH reflects enhanced visualization and hemostatic precision afforded by the robotic platform. The concomitant reduction in transfusion rates has important implications for donor safety and long-term outcomes, as perioperative blood transfusion is an established independent predictor of postoperative morbidity and prolonged hospitalization [32]. Despite improved hemostatic performance, no meaningful differences were observed in major vascular complications or hepatic artery thrombosis between the two techniques. These findings indicate comparable vascular safety profiles in high-expertise settings.

Operative time remains a key point of differentiation between the two approaches. Cheah et al. demonstrated that analysis of total operative time revealed clear learning curves for robotic living donor right hepatectomy, with Cumulative Sum Control Chart (CUSUM) peaks occurring at the 17th case in one center and the 9th case in another. Beyond these inflection points, mean operative time decreased significantly—from 603 to 438 min (p < 0.001) and from 532 to 418 min (p = 0.002), respectively [33]. These findings suggest that structured robotic training and proctorship programs may facilitate adoption, even in centers with limited prior experience in laparoscopic major hepatectomy, thereby enabling more rapid and scalable implementation across transplant programs [25, 34]. Consistent with broader experience in minimally invasive liver surgery, operative efficiency with robotic platforms improves as surgical teams accumulate experience, perioperative outcomes are maintained or further optimized; this is a trend well documented in high-volume centers with established robotic expertise.

Although robotic donor hepatectomy was associated with longer operative time in our pooled analysis, this finding should be interpreted within the context of early adoption and learning-curve effects rather than as an inherent limitation of the platform [35, 36]. In minimally invasive hepatectomy more broadly, operative duration typically improves with cumulative experience, refined case selection, and standardized team workflows, a pattern that has also been demonstrated in living donor surgery [37]. For pure laparoscopic donor right hepatectomy, CUSUM analyses indicate a prolonged learning phase of approximately 60 cases in one series [38], reflecting the technical complexity of major donor resections and the need to consolidate advanced laparoscopic skills and perioperative coordination. In contrast, donor-specific data for robotic living donor right hepatectomy suggest an earlier inflection in operative efficiency, with CUSUM peaks reported between 9 and 17 cases across specialized centers and a corresponding reduction in operative time thereafter [33]. Collectively, these observations support the interpretation that the longer operative times observed in comparative robotic cohorts may be driven by early implementation phases and case-mix differences rather than intrinsic inefficiency of the robotic approach. Nevertheless, because these learning-curve data are derived predominantly from high-volume expert programs, any inference regarding the relative “ease of adoption” of robotics should be framed as conditional upon structured training, monitored practice, and substantial institutional experience in both LDLT and minimally invasive liver surgery. Another notable finding was the longer second warm ischemia time observed in the robotic group. Although the difference was modest, prolonged warm ischemia has been associated with early graft dysfunction in some transplant settings and may reflect additional time required for graft extraction or team coordination during minimally invasive procurement. However, its clinical relevance remains uncertain, as recipient morbidity and mortality were not adversely affected in the pooled analysis.

Donor hepatectomy—particularly major resections—remains technically demanding, limiting widespread adoption of minimally invasive approaches and contributing to notable conversion rates to open surgery [39]. Although our pooled analysis did not demonstrate a statistically significant difference in conversion rates between robotic and laparoscopic approaches, sensitivity analysis excluding one outlier study (Rho et al., 2022) revealed a significantly lower conversion rate with R-LDH. Contemporary studies of minimally invasive major hepatectomy have similarly reported lower conversion rates with robotic compared with laparoscopic approaches [40, 41]. These findings suggest that the robotic platform may enable a greater proportion of complex major hepatectomies to be completed minimally invasively. Consistent with this, an international multicenter propensity score–matched analysis reported lower conversion rates in robotic right or extended right hepatectomy compared with laparoscopic procedures (8.6% vs. 17.7%; P = 0.01). However, when analysis was restricted to cases performed between 2015 and 2020 in centers with > 50 minimally invasive liver resections, this difference was no longer statistically significant (7.6% vs. 10.8%; P = 0.46). Furthermore, conversion rates for laparoscopic hepatectomy declined significantly over time (2008–2014 vs. 2015–2020), whereas robotic conversion rates remained stable [42].

The reduction in recipient biliary complications associated with robotic donor hepatectomy represents a key distinguishing finding. Kim et al. reported that robotic procurement was associated with significantly fewer multiple bile duct openings (26.8% vs. 54.9%; p = 0.001) and lower rates of major biliary complications (22.5% vs. 42.3%; p = 0.012). Late biliary complications (occurring after 90 days) were also significantly lower in the robotic group (11.3% vs. 23.9%; P = 0.047) [10]. Interpretation of these findings must consider the historical context of minimally invasive donor procurement. Early adoption of laparoscopic living donor hepatectomy occurred under substantial scrutiny, with outcomes heavily influenced by center experience, donor selection, and steep learning curves; a systematic review by Bekheit et al. concluded that laparoscopic procurement could achieve donor safety comparable to open surgery, but primarily within highly specialized settings during early dissemination. In contrast, contemporary robotic donor hepatectomy has generally been introduced in centers with established minimally invasive pathways and mature transplant infrastructure, and large prospective registry data have reported lower donor morbidity with robotics compared with laparoscopic or open approaches. Nonetheless, these comparisons remain susceptible to confounding by case selection, institutional volume, surgeon expertise, and temporal improvements in perioperative care; thus, the apparent safety advantage of robotics should be interpreted as reflecting both platform-related technical facilitation and late-stage adoption in high-performing systems, rather than universal superiority across all centers [43].

Beyond visualization, additional technical advantages of the robotic platform—including tremor filtration, improved ergonomics, and stable three-dimensional optics—may contribute to reduced biliary complications [44, 45]. While ICG fluorescence is also available in laparoscopic surgery, robotic systems may allow more seamless integration of imaging within the operative workflow and greater precision during critical biliary steps [46, 47]. Collectively, these factors may reduce inadvertent bile duct injury and improve anatomical identification, although the relative contributions of platform features versus surgeon experience and standardized technique cannot be fully disentangled in observational studies.

Cost and resource implications are central to the real-world adoption of robotic donor hepatectomy. Although our meta-analysis demonstrates clinically meaningful advantages of R-LDH—including reduced blood loss, lower transfusion rates, and decreased overall donor morbidity—robotic procurement typically entails higher fixed and procedural costs related to platform acquisition, maintenance, instrumentation, and operating room utilization. Additionally, longer operative times observed in our pooled analysis represent a significant cost driver. Evidence from the broader hepatectomy literature suggests that laparoscopic liver resection is generally the most cost-effective minimally invasive strategy [48], whereas robotic liver resection is usually more expensive despite comparable or favorable outcomes in selected settings. Consequently, while reductions in complications or length of stay may offset costs in some high-volume centers, the net economic value of robotic donor hepatectomy is likely center- and system-dependent and cannot be reliably inferred without donor-specific cost analyses [49].

From an implementation perspective, our findings indicate that robotic donor hepatectomy can be safely integrated in appropriately resourced programs and may offer meaningful donor benefits, particularly lower blood loss, reduced transfusion requirements, and decreased overall morbidity—while maintaining comparable rates of major complications. These advantages are most plausible in high-volume LDLT centers with standardized donor selection pathways, established perioperative protocols, and multidisciplinary expertise in complex hepatobiliary surgery [23, 50]. However, longer operative times, center-dependent learning curves, and the absence of pooled economic data suggest that robotic adoption should not be characterized as universally “easier” or inherently superior. Rather, robotics may be best viewed as an enabling platform for centers pursuing minimally invasive donor procurement within structured training frameworks, clear case-selection criteria, and continuous outcome monitoring—particularly for biliary endpoints, where heterogeneity in definitions warrants cautious interpretation. Future research should prioritize standardized reporting of donor-specific costs, learning curve metrics, and long-term biliary and functional outcomes.

### Strengths and limitations

This study has several notable strengths. First, it represents the largest comparative analysis of robotic versus laparoscopic living donor hepatectomy to date, including over 15,000 donors across seven contemporary studies published between 2022 and 2025. Second, we evaluated both donor and recipient outcomes, providing a comprehensive assessment of the impact of surgical modality across the entire transplantation process, addressing an important gap in prior reviews that primarily focused on donor outcomes.

However, several limitations must be acknowledged. First, all included studies were observational; no randomized controlled trials directly comparing R-LDH and L-LDH are currently available. An additional consideration is the inclusion of studies reporting different graft types, including right, left, and left lateral hepatectomies. These procedures vary in technical complexity and risk profile and may contribute to clinical heterogeneity across studies. Because several large comparative studies reported outcomes across multiple graft types, these studies were included to reflect contemporary clinical practice in living donor hepatectomy. Nevertheless, future investigations with graft-specific stratification may help clarify whether procedural complexity modifies the relative benefits of robotic assistance.

Second, substantial heterogeneity was observed in operative time, blood loss, and length of hospital stay, likely reflecting variations in center volume, surgeon experience, and perioperative protocols. Sensitivity analysis showed that excluding the large LDLT registry study reversed the pooled estimate for donor major morbidity, suggesting a higher risk with the robotic approach. This finding should be interpreted cautiously, as the registry represents the largest donor cohort and reflects outcomes from multiple high-volume centers. In addition, Doi plot analysis demonstrated major asymmetry (LFK index >  ± 2) across several outcomes, indicating potential small-study effects whereby smaller studies may preferentially report favorable robotic outcomes. Together, these factors suggest that some observed advantages of robotic donor hepatectomy should be interpreted cautiously and warrant confirmation in larger prospective or registry-based studies.

Third, the majority of included studies originated from high-volume centers in East Asia with extensive experience in both LDLT and minimally invasive surgery, potentially limiting generalizability to lower-volume centers or other regions. Fourth, we did not extract or pool economic outcomes, and cost reporting in donor hepatectomy remains sparse and heterogeneous; therefore, no conclusions regarding cost-effectiveness can be drawn. Fifth, definitions and reporting of biliary complications were not fully standardized across studies—often representing composite endpoints—contributing to heterogeneity, particularly in donor analyses. Even in propensity score–matched studies, residual confounding related to case selection, anatomical complexity, and institutional pathways may persist. Finally, long-term donor-reported outcomes, including quality of life, psychological impact, and late complications, remain underreported.

## Conclusion

Robotic living donor hepatectomy appears to offer meaningful perioperative benefits compared with the laparoscopic approach, including reduced blood loss, lower transfusion requirements, decreased overall donor morbidity, and fewer recipient biliary complications, although these advantages are accompanied by longer operative duration. Evidence derived from large multicenter observational cohorts and propensity-matched analyses supports the hypothesis that enhanced visualization and instrument articulation may improve precision during hilar and parenchymal dissection. However, current data remain largely observational, with notable inter-center variability and limited reporting of long-term donor-centered outcomes. Well-designed prospective investigations and comprehensive longitudinal registries are essential to clarify the durability of benefit and to guide optimal integration of robotic platforms into living donor liver transplantation programs worldwide.

## Supplementary Information

Below is the link to the electronic supplementary material.

Supplementary file1

Supplementary file2

Supplementary file3

## Data Availability

All data analyzed in this study are derived from previously published sources and are available within the article and its supplementary materials. Additional details regarding extracted datasets or analytical procedures may be obtained from the corresponding author upon reasonable request.
