# Correction: From laparoscopy to robotics in living donor hepatectomy: a systematic review and meta-analysis of comparative outcomes

**DOI:** 10.1007/s11701-026-03670-5

**Published:** 2026-07-20

**Authors:** Mohamed Al Sayed, Mahmoud Albashier, Hashem Altabbaa, Ahmad Omar Saleh, Abdalla M. Hadhoud, Mahmoud Ashraf Hussein, Abedalrahman Yazan Aljarrah, Menatalla M. Abdelrazek, Mohamed Bekheit

**Affiliations:** 1 Department of Upper Gastrointestinal and Liver Surgery, Unit 2, Alexandria Main University Hospital, Alexandria University, Alexandria, Egypt; 2 Department of Surgery, Faculty of Medicine, Alexandria University, Alexandria, Egypt; 3 The Research Papyrus Lab, Alexandria, Egypt; 4 Faculty of Medicine, Damietta University, New Damietta, Egypt; 5 Al-Basheer-Hospital, Amman, Jordan; 6 Faculty of Medicine, The University of Jordan, Amman, Jordan; 7 Department of Surgery, University of Cincinnati, Cincinnati, OH USA; 8 Faculty of Medicine, Capital University, Cairo, Egypt; 9 Princess Basma Teaching Hospital, Ministry of Health, Irbid, Jordan; 10 Alexandria Main University Hospital, Alexandria University, Alexandria, Egypt; 11 School of Medicine, University of Aberdeen, Aberdeen, UK

**Correction: Journal of Robotic Surgery (2026) 20:500**

10.1007/s11701-026-03360-2

In the original version of the article, the author’s name Mohamed Bekheit was incorrectly written as Mohamed Bakhiet.

