# Supplementary material for: From laparoscopy to robotics in living donor hepatectomy: a systematic review and meta-analysis of comparative outcomes
Source: J Robot Surg. 2026 May 14;20(1):500. doi: 10.1007/s11701-026-03360-2 (PMC13176131; doi:10.1007/s11701-026-03360-2)
Supplement: Supplementary file 1 — Supplementary file1 [file 11701_2026_3360_MOESM1_ESM.docx]

# **Concepts and MeSH Terms**

## **Concept one**

("Liver Transplantation" OR "Hepatectomy" OR "Liver Transplantation" OR "living donor liver transplantation" OR "living donor liver transplant" OR LDLT OR "living donor right hepatectomy" OR "donor hepatectomy" OR "right hepatectomy" OR "right hepatic lobectomy" OR "right lobe hepatectomy")

## **Concept two**

("Robotic Surgical Procedures" OR "Robotics" OR robot* OR "robotic" OR "robotic-assisted" OR "robot-assisted" OR "robotic hepatectomy")

## **Concept three**

(laparoscopic OR "laparoscopic surgery" OR "laparoscopic donor hepatectomy" OR conventional)

# **Final Search Strategy**

(("Liver Transplantation" OR "Hepatectomy" OR "Liver Transplantation" OR "living donor liver transplantation" OR "living donor liver transplant" OR LDLT OR "living donor right hepatectomy" OR "donor hepatectomy" OR "right hepatectomy" OR "right hepatic lobectomy" OR "right lobe hepatectomy") AND ("Robotic Surgical Procedures" OR "Robotics" OR robot* OR "robotic" OR "robotic-assisted" OR "robot-assisted" OR "robotic hepatectomy") AND (laparoscopic OR "laparoscopic surgery" OR "laparoscopic donor hepatectomy" OR conventional))

## **PubMed: 563**

(("Liver Transplantation"[Mesh] OR "Hepatectomy"[Mesh] OR "Liver Transplantation"[Title/Abstract] OR "living donor liver transplantation"[Title/Abstract] OR "living donor liver transplant"[Title/Abstract] OR LDLT[Title/Abstract] OR "living donor right hepatectomy"[Title/Abstract] OR "donor hepatectomy"[Title/Abstract] OR "right hepatectomy"[Title/Abstract] OR "right hepatic lobectomy"[Title/Abstract] OR "right lobe hepatectomy"[Title/Abstract]) AND ("Robotic Surgical Procedures"[Mesh] OR "Robotics"[Mesh] OR robot*[Title/Abstract] OR "robotic"[Title/Abstract] OR "robotic-assisted"[Title/Abstract] OR "robot-assisted"[Title/Abstract] OR "robotic hepatectomy"[Title/Abstract]) AND (laparoscopic[Title/Abstract] OR "laparoscopic surgery"[Title/Abstract] OR "laparoscopic donor hepatectomy"[Title/Abstract] OR conventional[Title/Abstract]))

## **Scopus: 947**

TITLE-ABS-KEY ( ( ( "Liver Transplantation" OR "Hepatectomy" OR "Liver Transplantation" OR "living donor liver transplantation" OR "living donor liver transplant" OR LDLT OR "living donor right hepatectomy" OR "donor hepatectomy" OR "right hepatectomy" OR "right hepatic lobectomy" OR "right lobe hepatectomy" ) AND ( "Robotic Surgical Procedures" OR "Robotics" OR robot* OR "robotic" OR "robotic-assisted" OR "robot-assisted" OR "robotic hepatectomy" ) AND (laparoscopic OR "laparoscopic surgery" OR "laparoscopic donor hepatectomy" OR conventional)))

## **WOS: 275**

TS=("living donor" OR LDLT OR "living donor liver transplantation" OR "donor hepatectomy" OR "right hepatectomy") AND TS=(robot* OR robotic OR "robotic-assisted" OR "robot-assisted") AND TS=(laparoscopic OR "laparoscopic surgery" OR "laparoscopic donor hepatectomy" OR conventional)

## **Cochrane: 11**

("living donor" OR LDLT OR "living donor liver transplantation" OR "donor hepatectomy" OR "right hepatectomy" OR "right hepatic lobectomy") AND (robot* OR robotic OR "robotic-assisted" OR "robot-assisted" OR "robotic surgical") AND (laparoscopic OR "laparoscopic surgery" OR "laparoscopic donor hepatectomy" OR conventional)

## **Embase: 609**

(('liver transplantation'/exp OR 'hepatectomy'/exp OR 'liver transplantation':ti,ab OR 'living donor liver transplantation':ti,ab OR 'living donor liver transplant':ti,ab OR LDLT:ti,ab OR 'living donor right hepatectomy':ti,ab OR 'donor hepatectomy':ti,ab OR 'right hepatectomy':ti,ab OR 'right hepatic lobectomy':ti,ab OR 'right lobe hepatectomy':ti,ab) AND ('robotic surgery'/exp OR 'robotics'/exp OR robot*:ti,ab OR 'robotic':ti,ab OR 'robotic-assisted':ti,ab OR 'robot-assisted':ti,ab OR 'robotic hepatectomy':ti,ab) AND (laparoscopic:ti,ab OR 'laparoscopic surgery':ti,ab OR 'laparoscopic donor hepatectomy':ti,ab OR conventional:ti,ab))
