# Supplementary material for: From laparoscopy to robotics in living donor hepatectomy: a systematic review and meta-analysis of comparative outcomes
Source: J Robot Surg. 2026 May 14;20(1):500. doi: 10.1007/s11701-026-03360-2 (PMC13176131; doi:10.1007/s11701-026-03360-2)
Supplement: Supplementary file 2 — Supplementary file2 [file 11701_2026_3360_MOESM2_ESM.docx]

**Electronic Supplementary Material 2 (ESM2)**
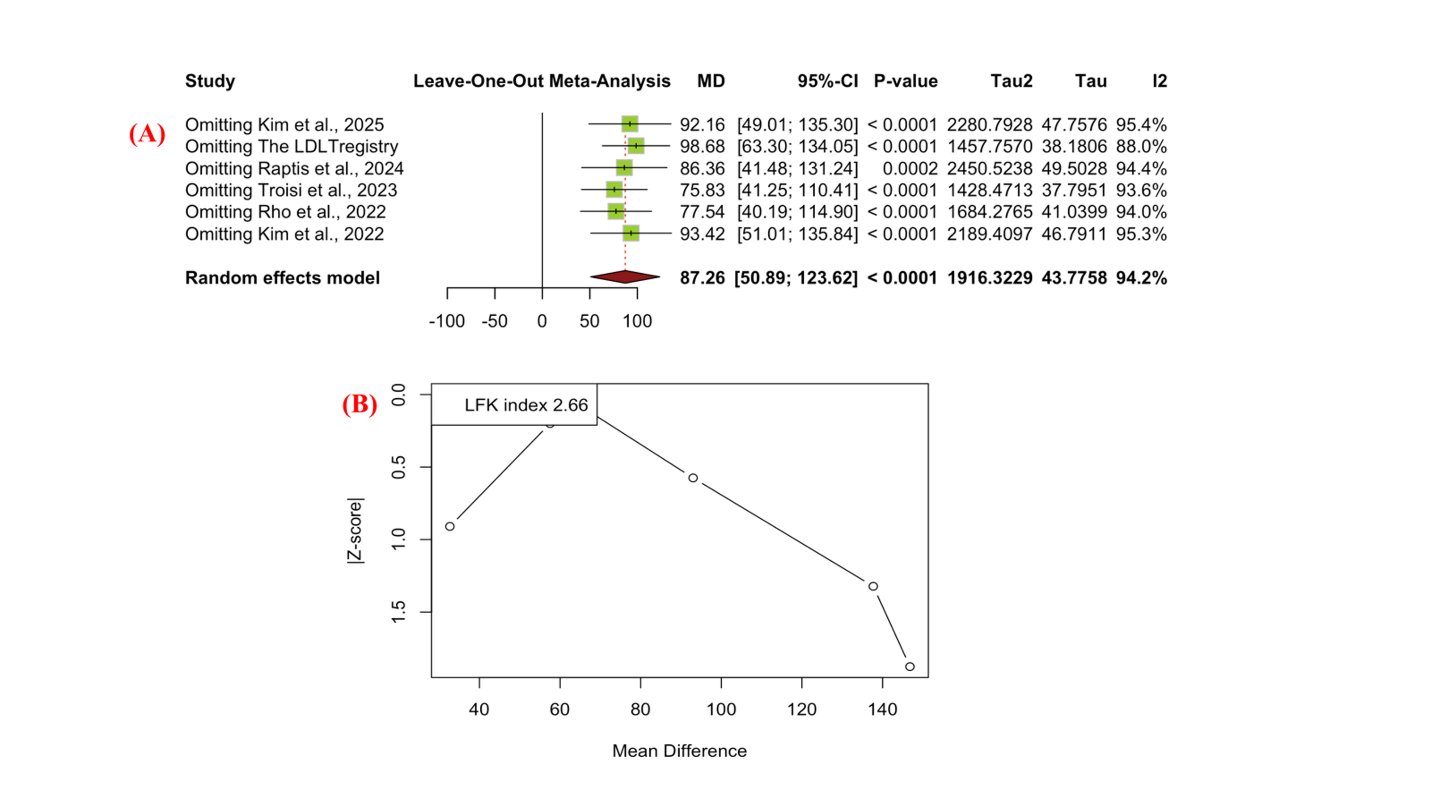


**(Fig. S2.1) Leave-one-out sensitivity analysis and Doi plot evaluating potential publication bias for operative time.**


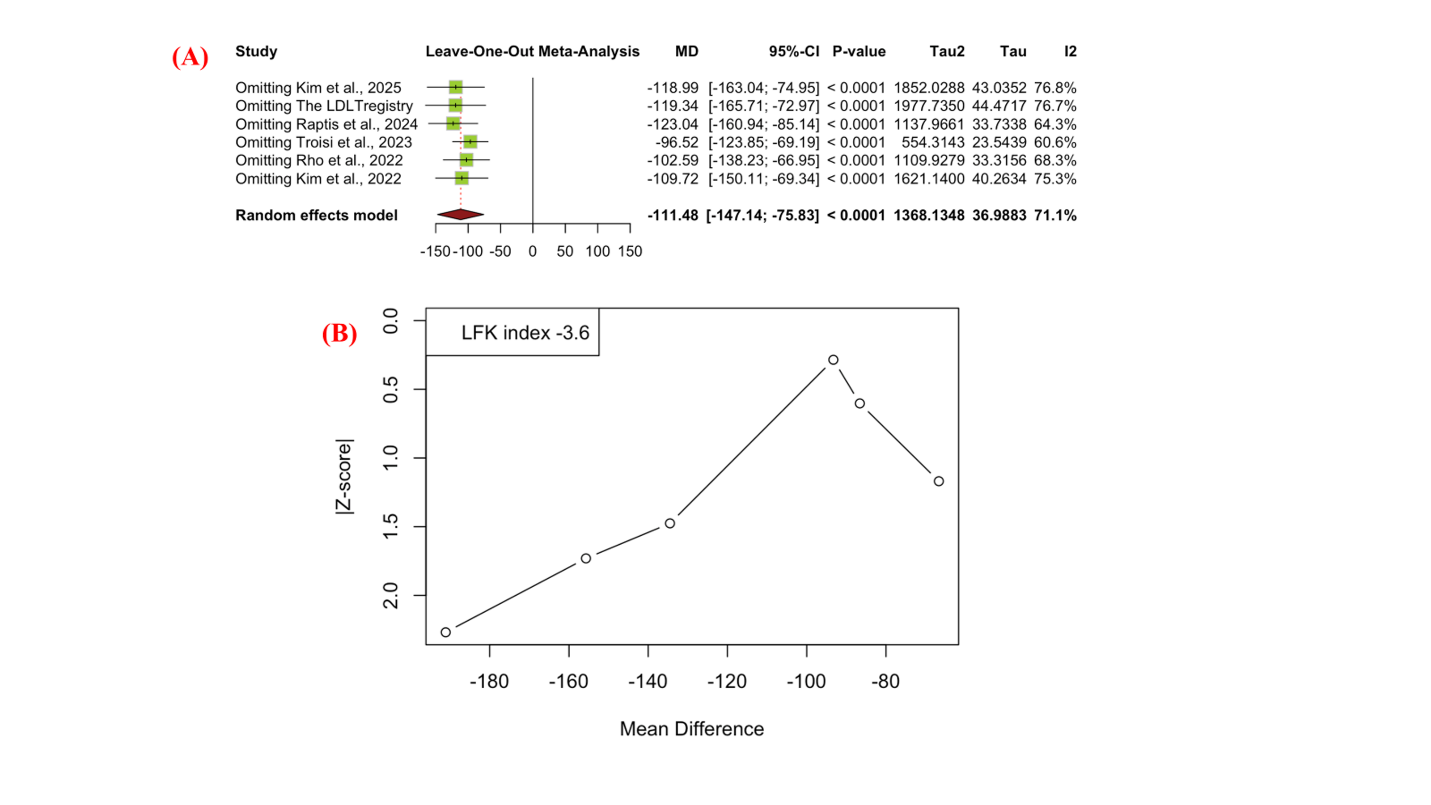


**(Fig. S2.2) Leave-one-out sensitivity analysis and Doi plot evaluating potential publication bias for estimated blood loss.**


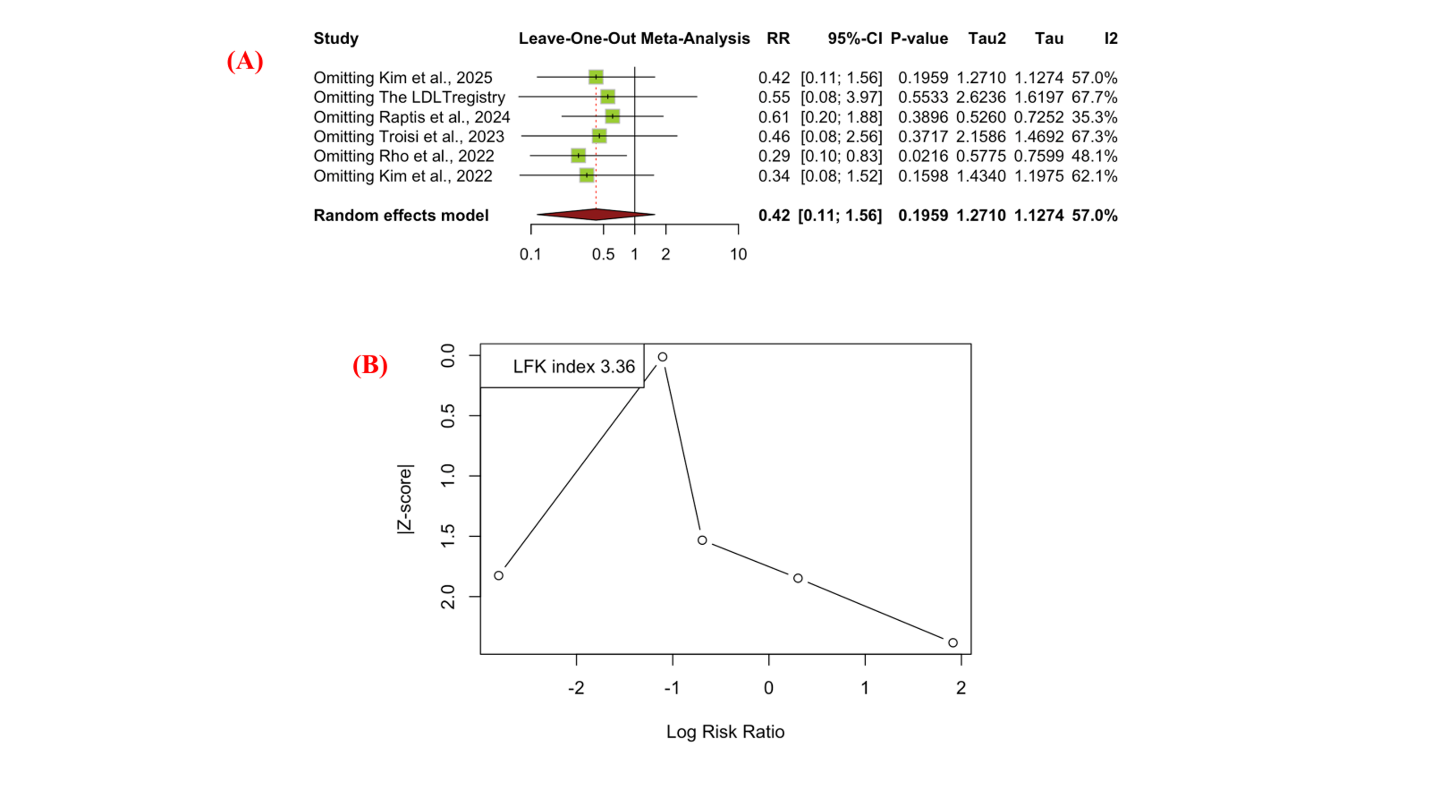


**(Fig. S2.3) Leave-one-out sensitivity analysis and Doi plot evaluating potential publication bias for conversion to open hepatectomy.**

**
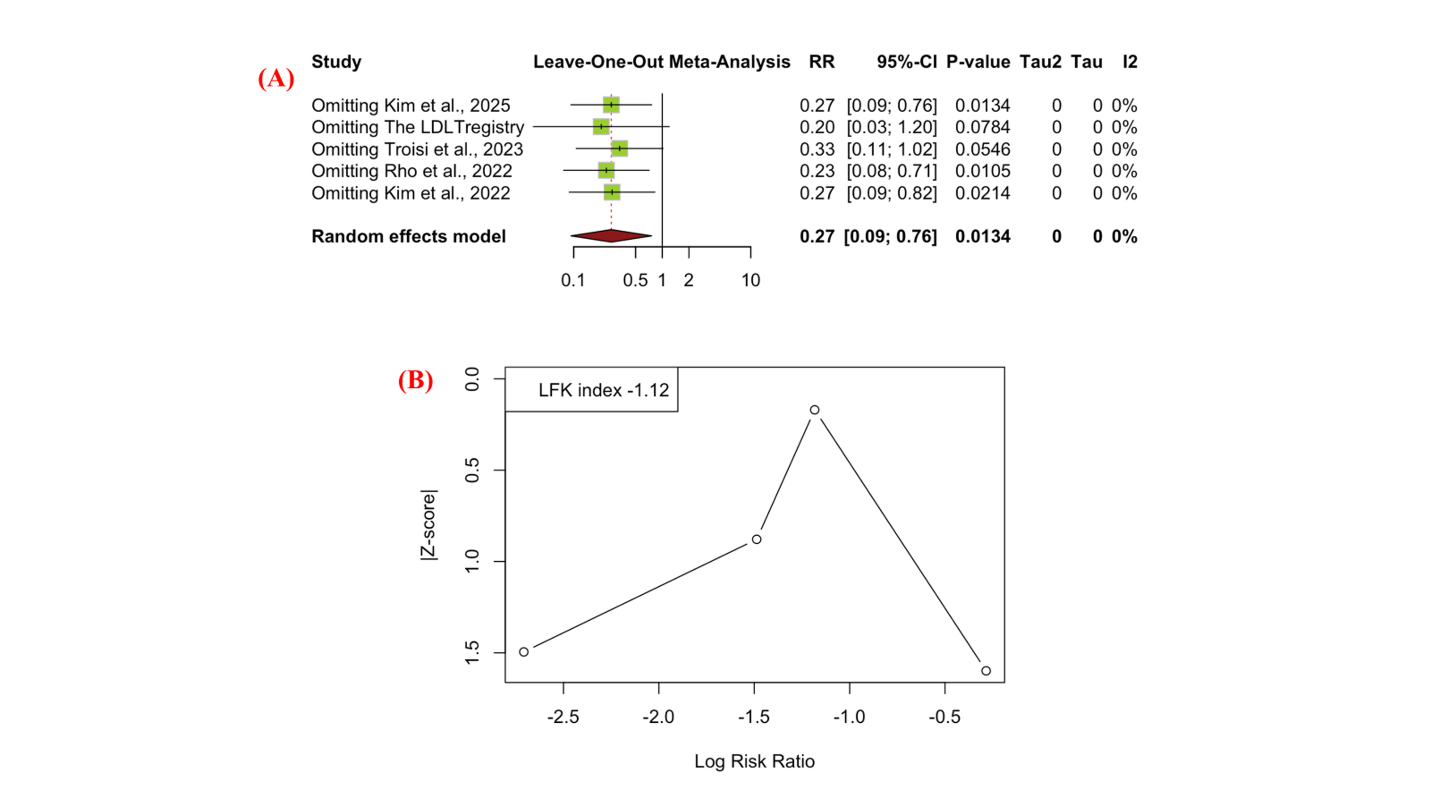
**

**(Fig. S2.4) Leave-one-out sensitivity analysis and Doi plot evaluating potential publication bias for blood transfusion.**

**
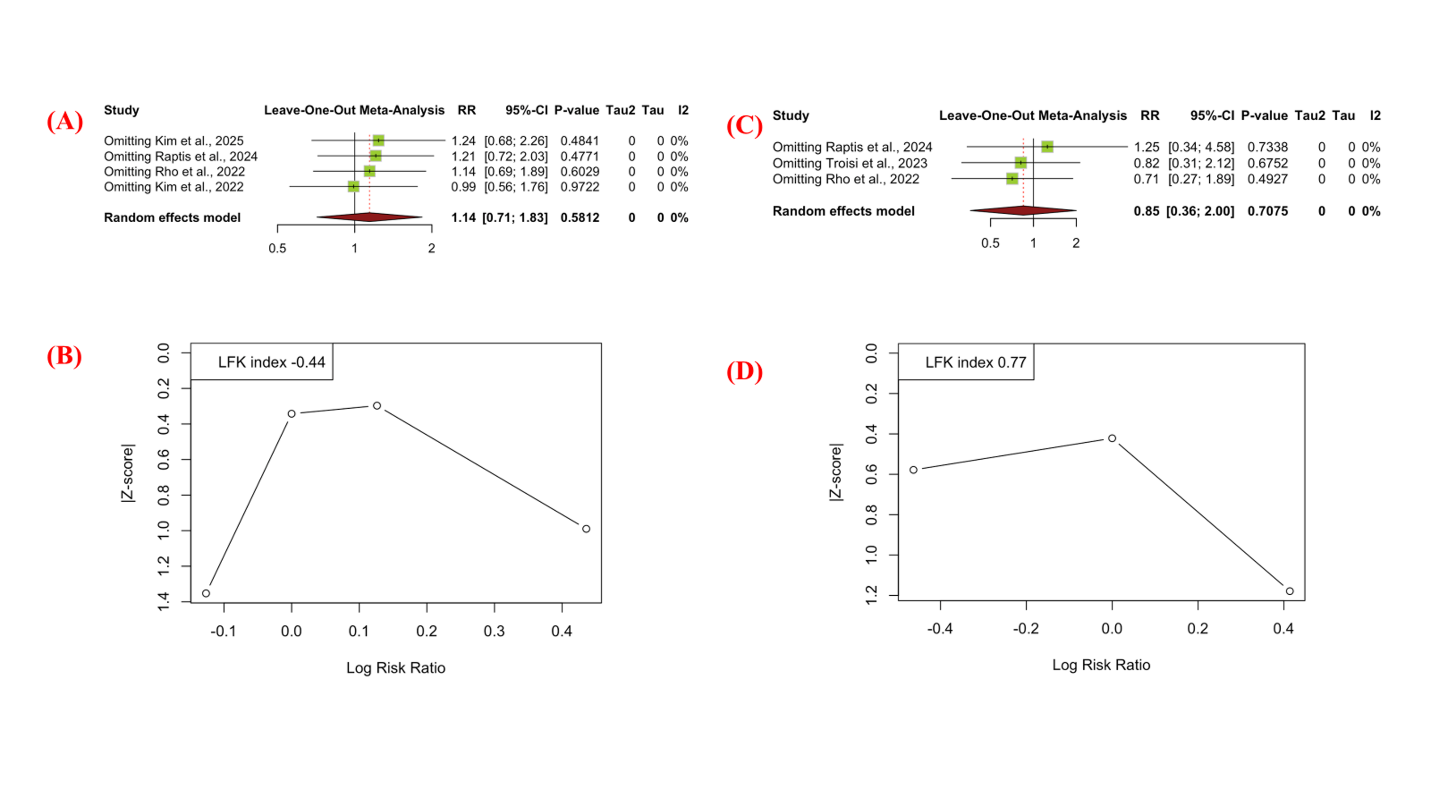
**

**(Fig. S2.5) Leave-one-out sensitivity analysis and Doi plot evaluating potential publication bias for overall vascular complications and Hepatic Artery Thrombosis (HAT).**

**
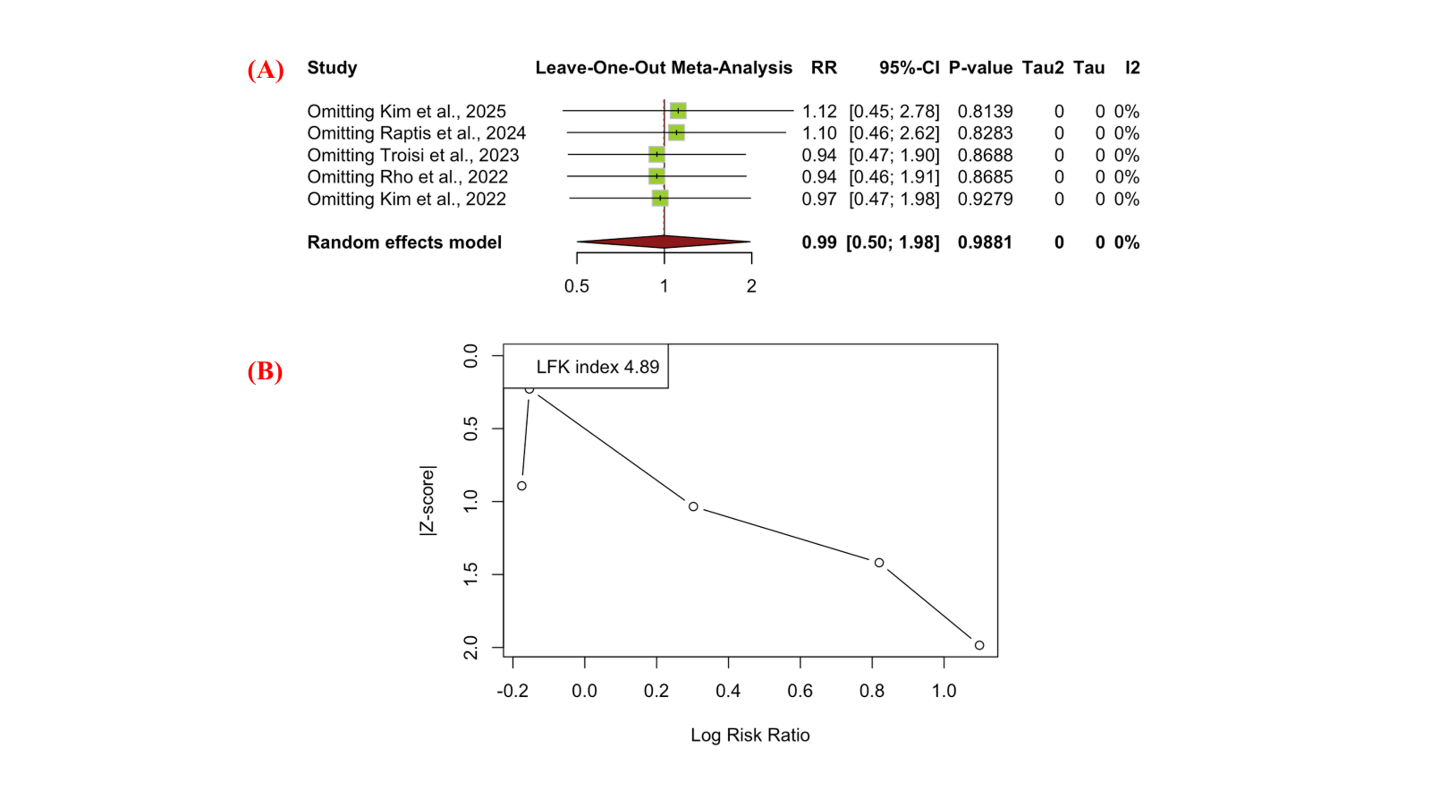
**

**(Fig. S2.6) Leave-one-out sensitivity analysis and Doi plot assessing potential publication bias regarding rates of mortality.**


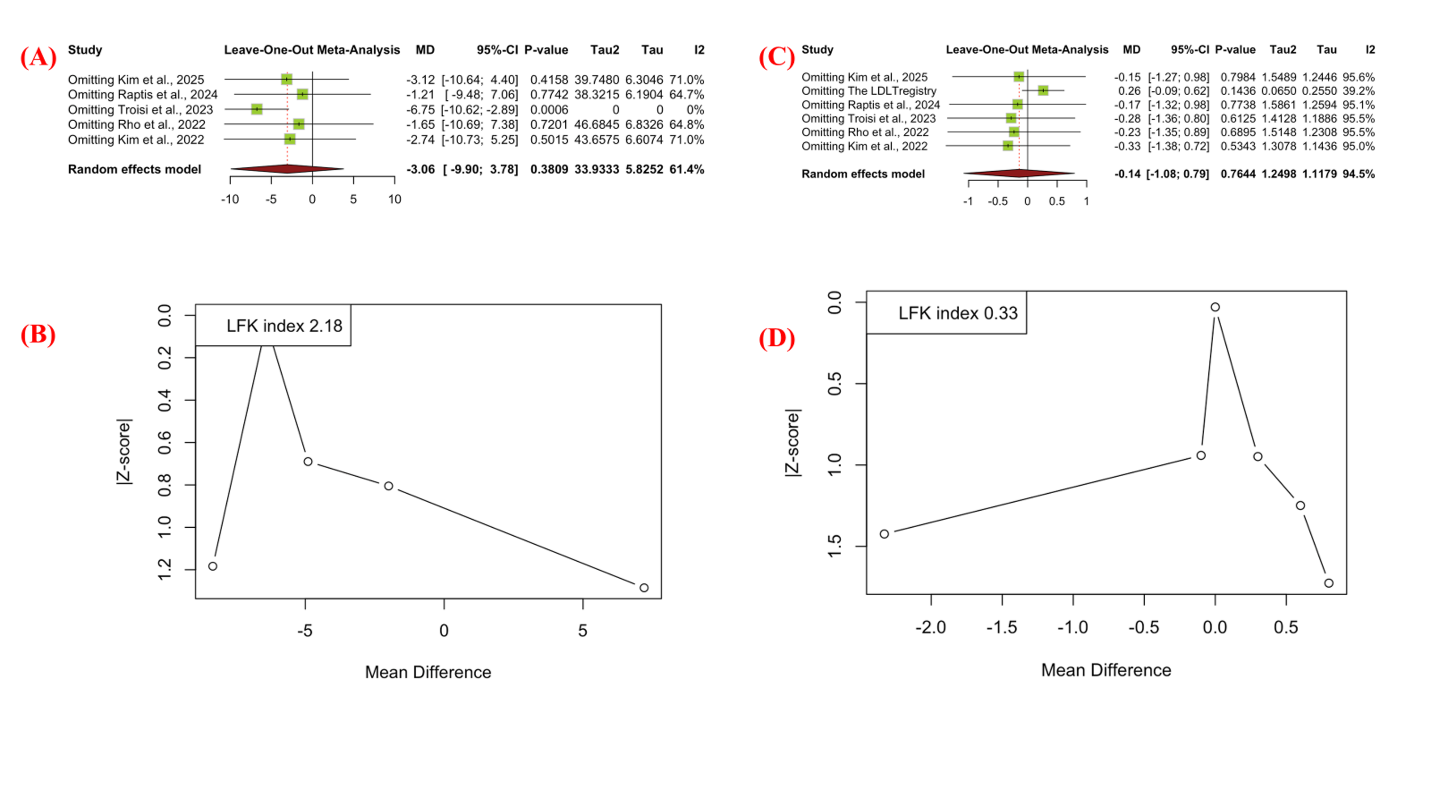


**(Fig. S2.7) Leave-one-out sensitivity analysis and Doi plot evaluating potential publication bias for hospital stay in recipients and donors.**


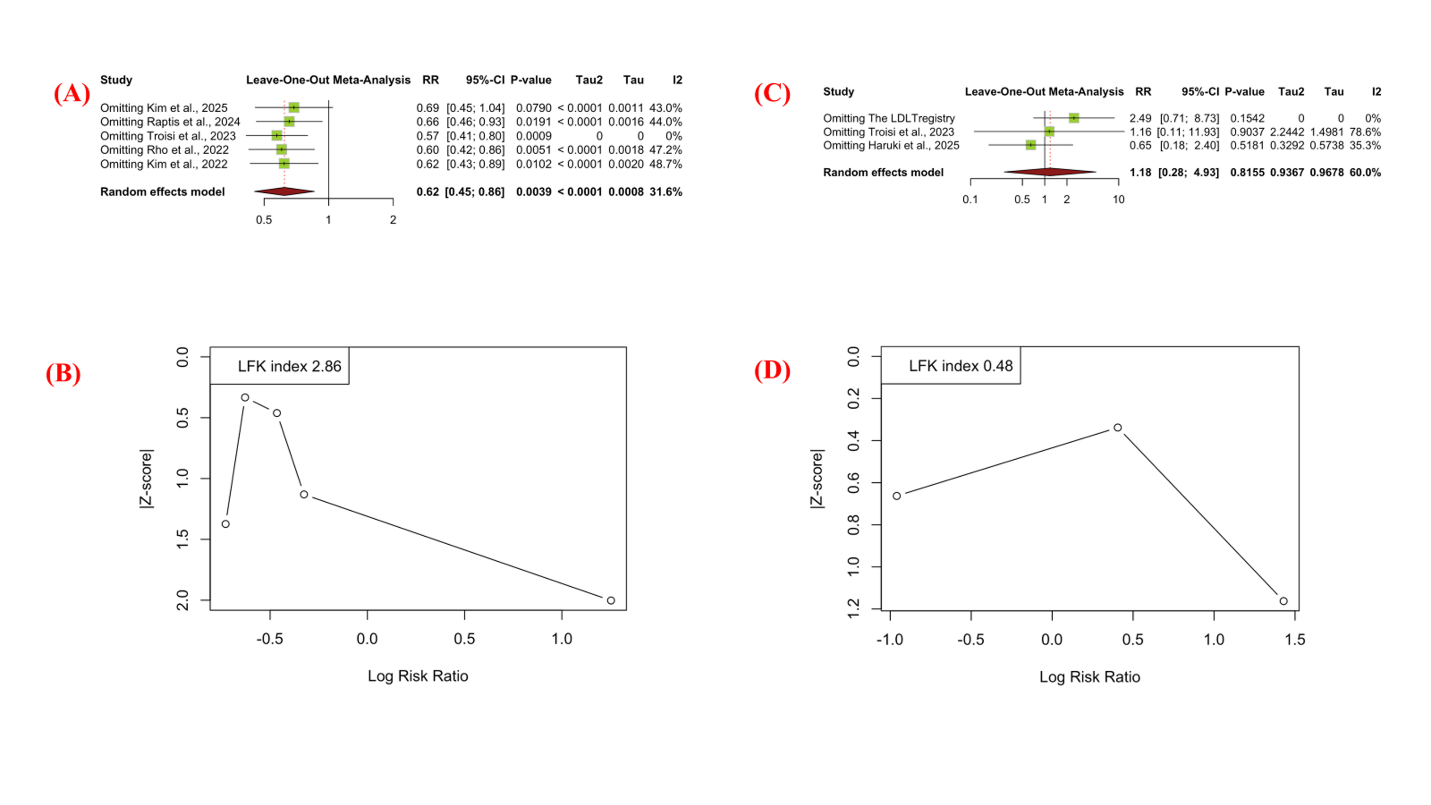


**(Fig. S2.8) Leave-one-out sensitivity analysis and Doi plot assessing potential publication bias regarding overall biliary complications in recipients and biliary leakage in donors.**


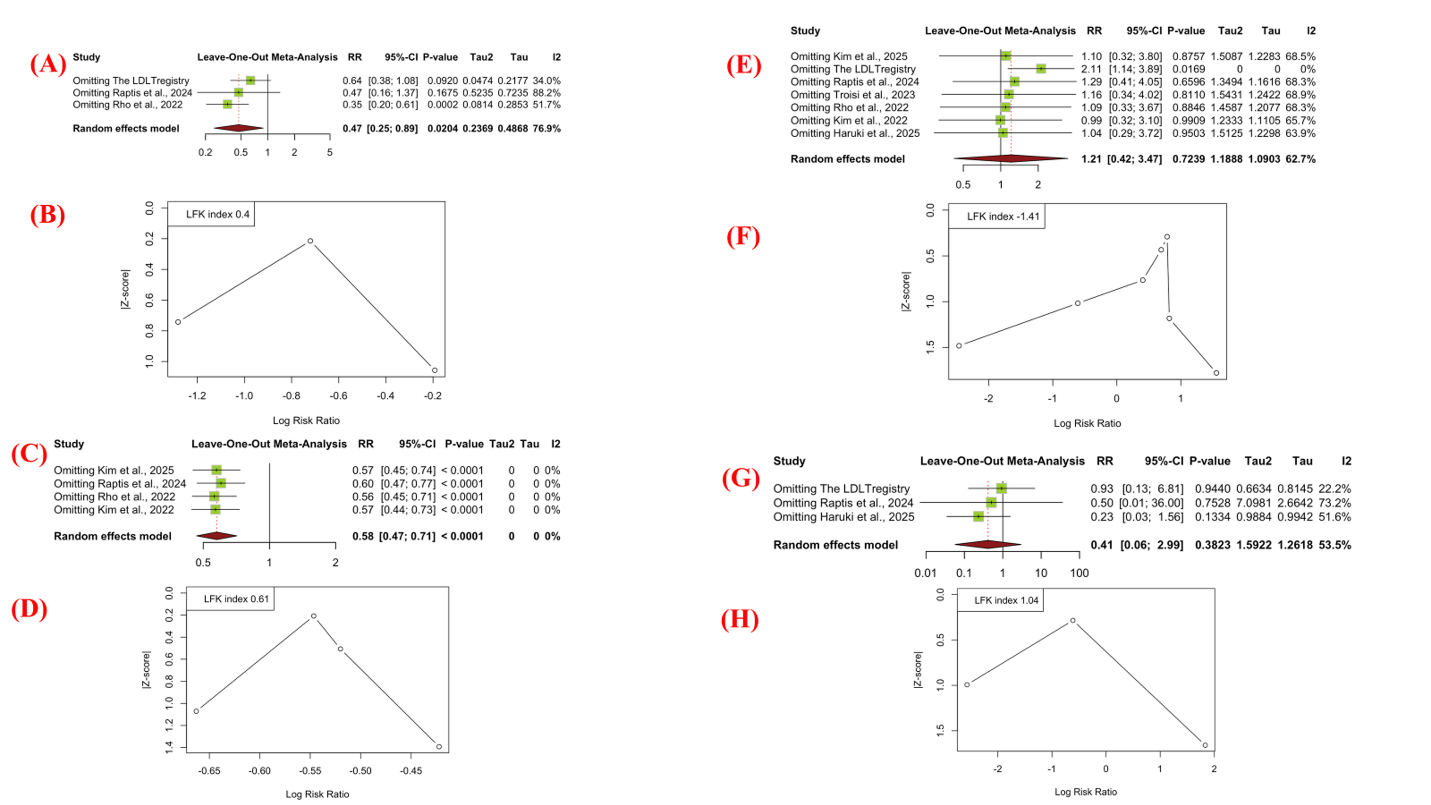


**Figure S2.9 illustrates the leave-one-out sensitivity analyses and Doi plots evaluating the robustness of the pooled estimates and the potential for publication bias with respect to overall donor morbidity, major morbidity in both donors and recipients, and donor infectious complications.**

**
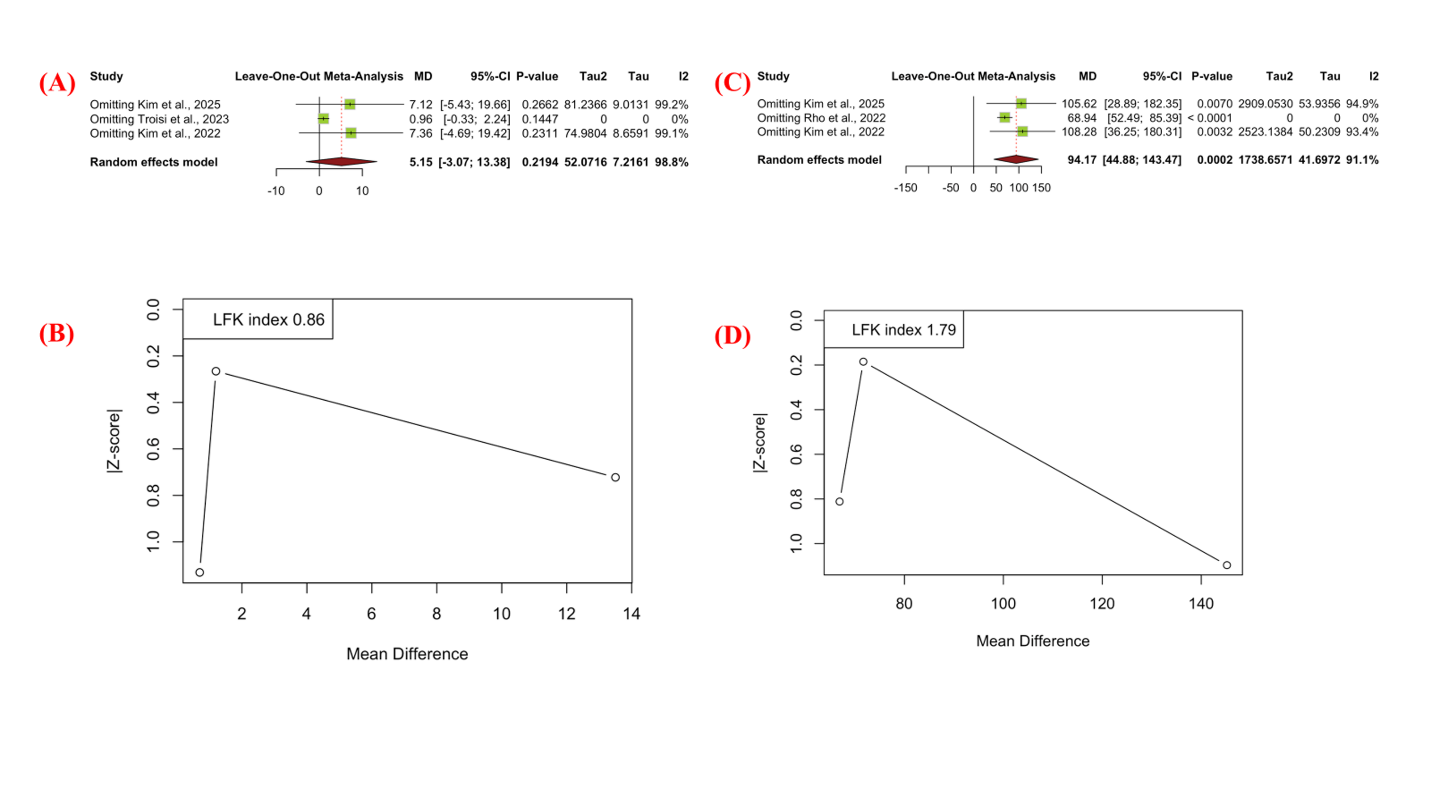
**

**Figure S2.10 displays the leave-one-out sensitivity analyses and Doi plots, which evaluate the robustness of the pooled estimates and examine the potential for publication bias related to first warm ischemia times and graft-out time.**

**
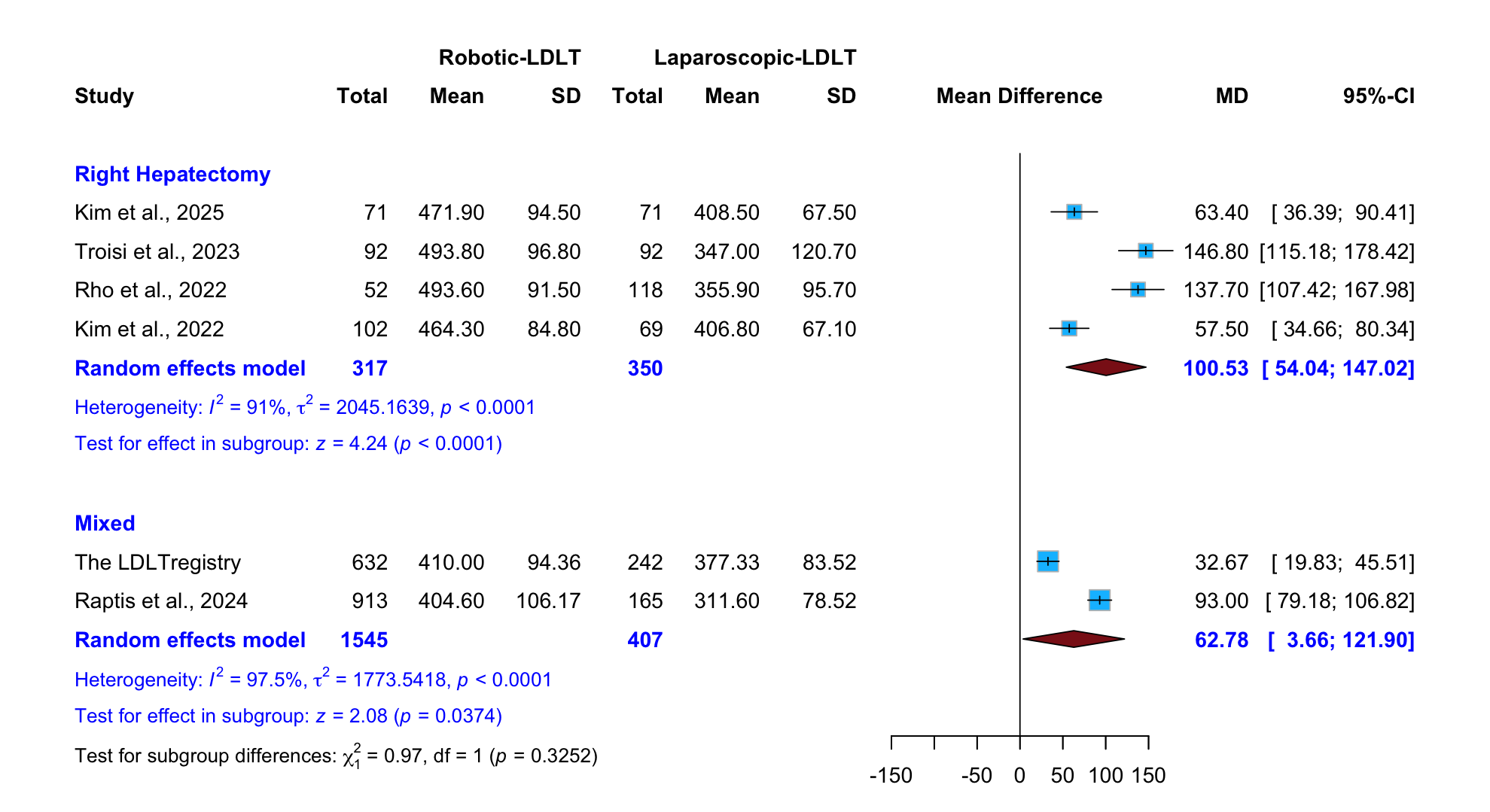
**

**Fig. S2.11 – Subgroup analysis by graft type for operative time.**

**
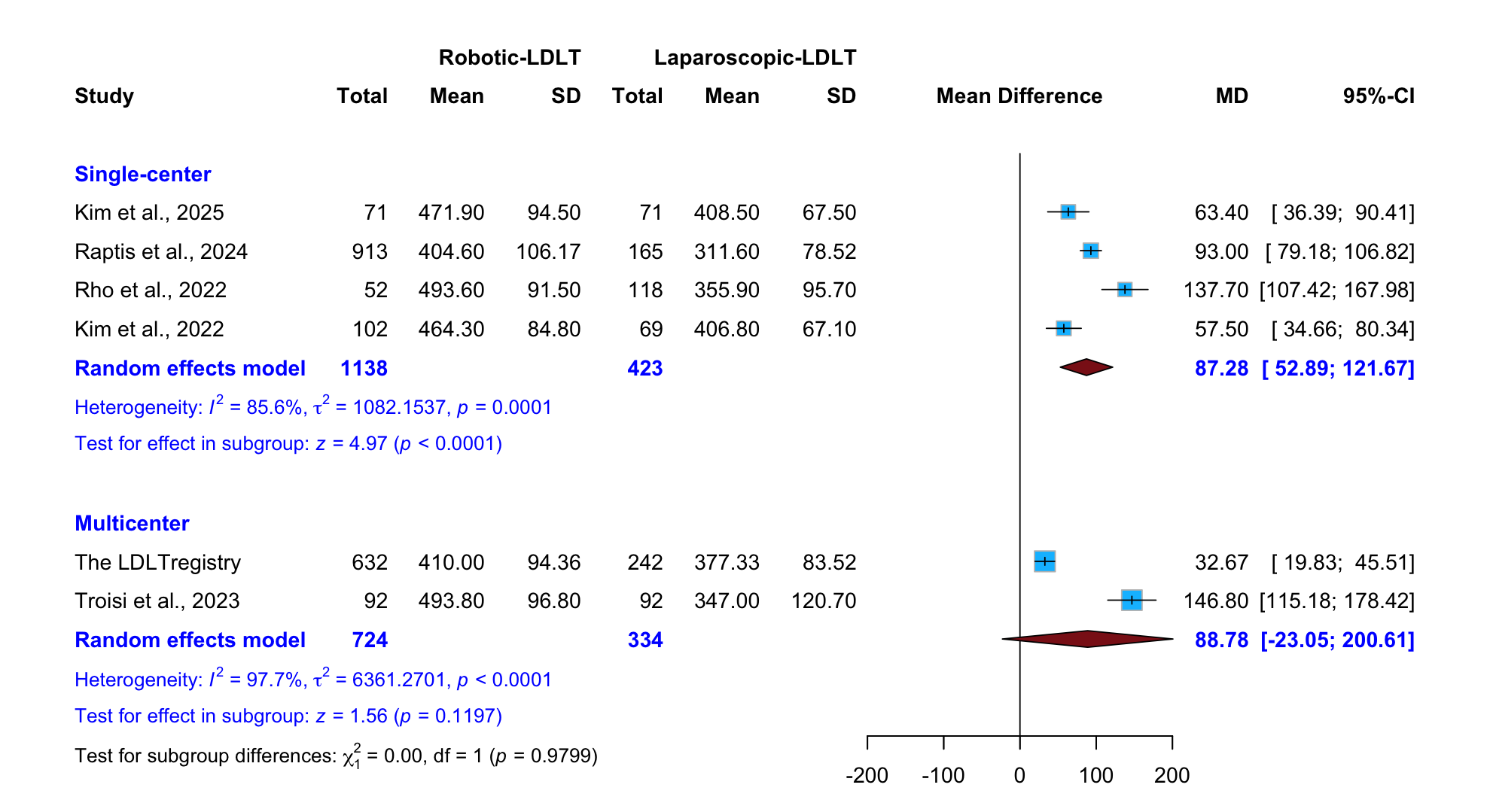
**

**Fig. S2.12 – Subgroup analysis by study design for operative time**

**
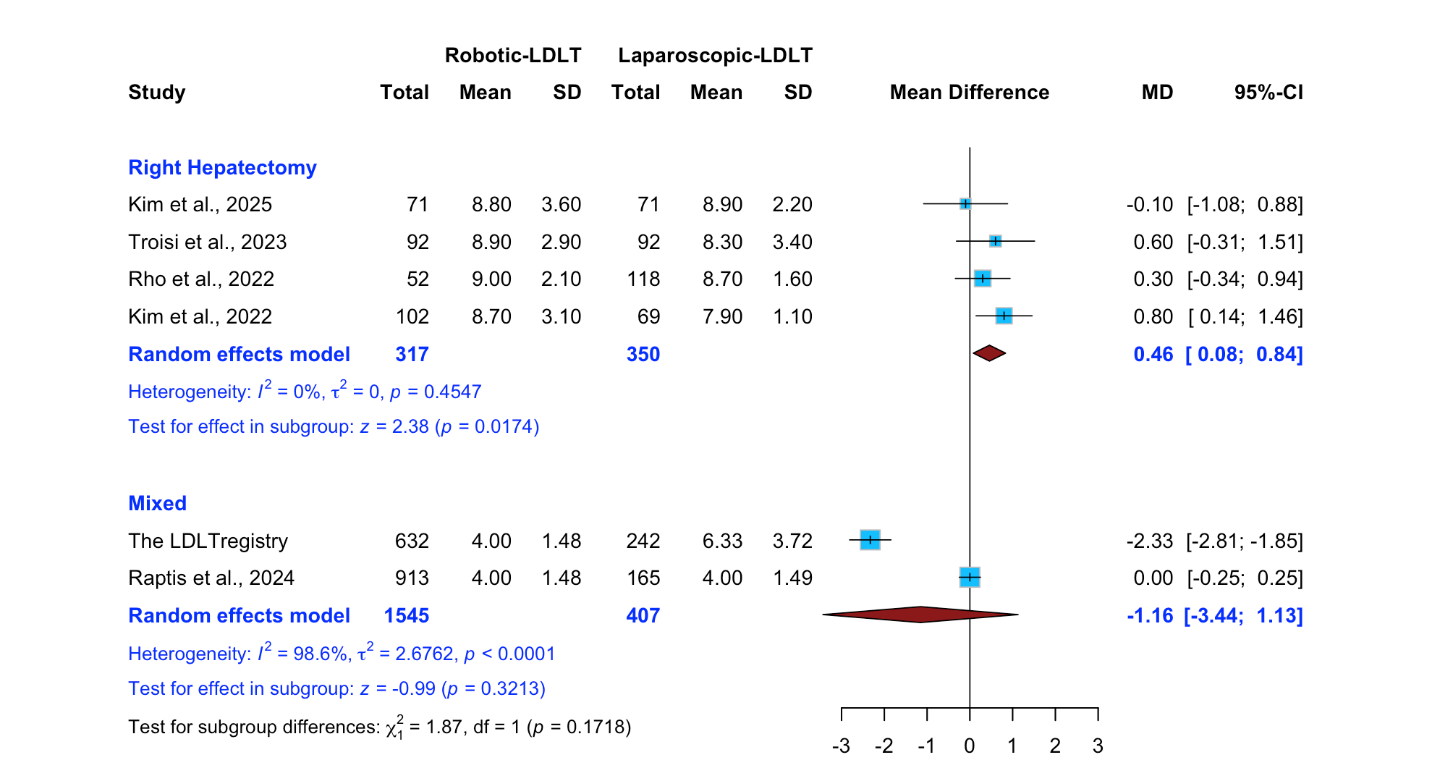
**

**Fig. S2.13 – Subgroup analysis of donor hospital stay according to graft type.**

**
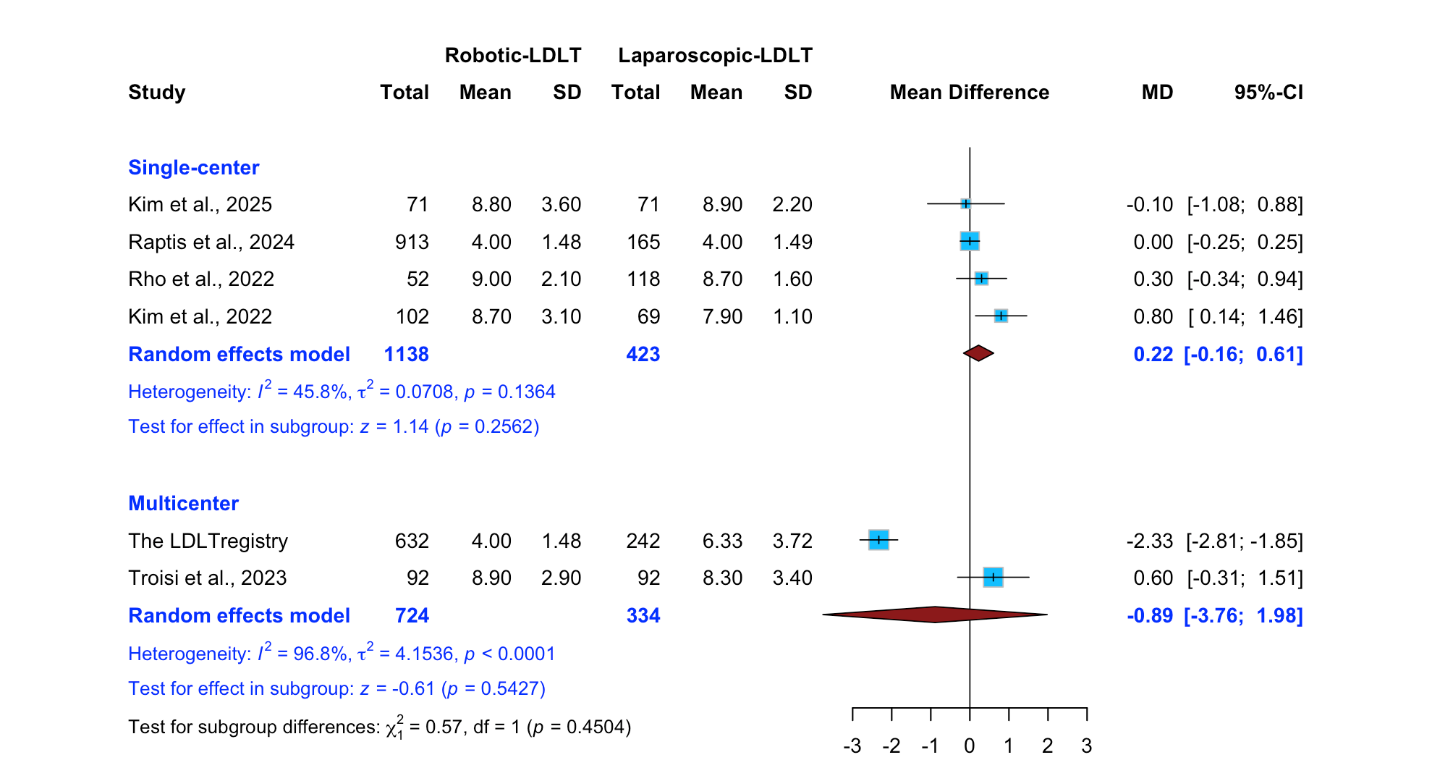
**

**Fig. S2.14 – Subgroup analysis of donor hospital stay according to study design.**

**
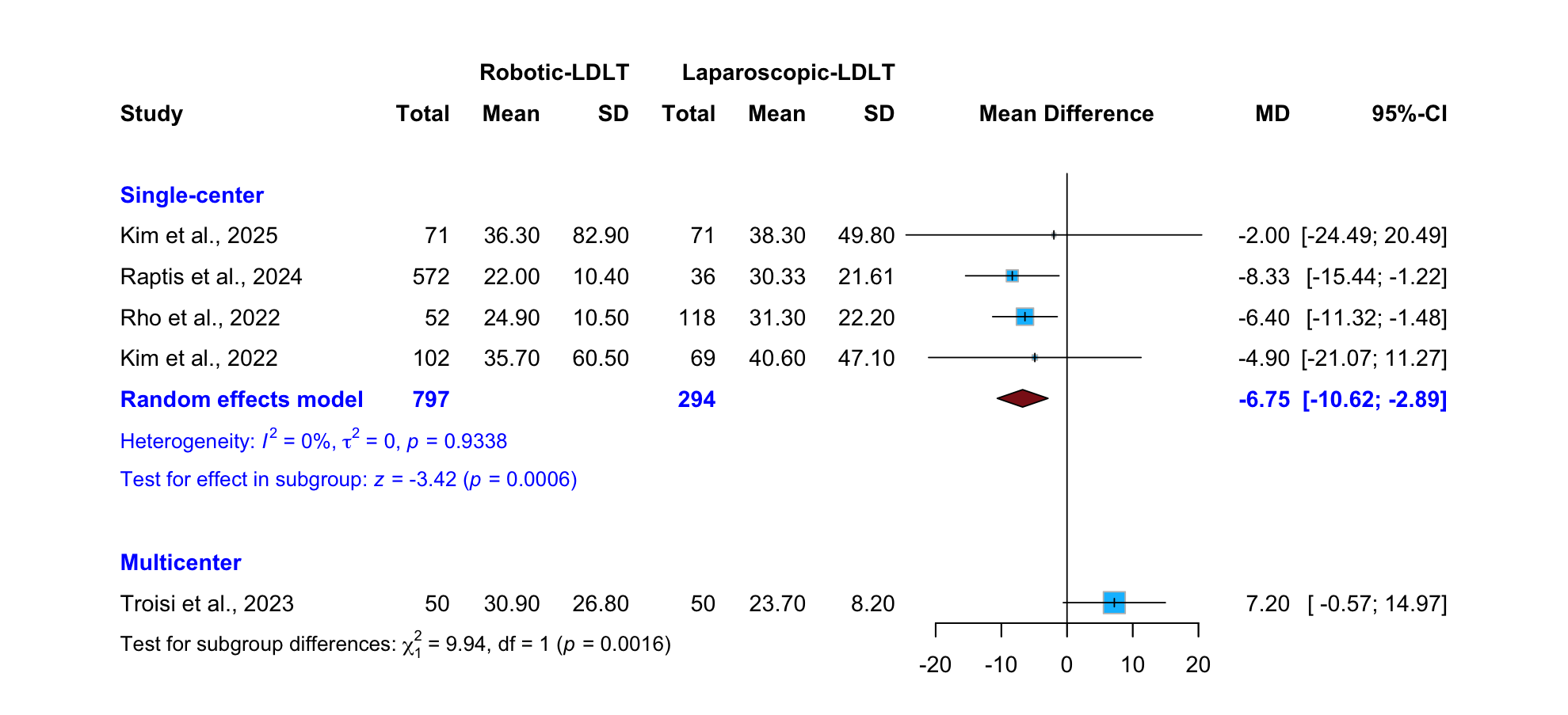
**

**Fig. S2.15 – Subgroup analysis of recipient hospital stay according to study design.**
