# Supplementary material for: From laparoscopy to robotics in living donor hepatectomy: a systematic review and meta-analysis of comparative outcomes
Source: J Robot Surg. 2026 May 14;20(1):500. doi: 10.1007/s11701-026-03360-2 (PMC13176131; doi:10.1007/s11701-026-03360-2)
Supplement: Supplementary file 3 — Supplementary file3 [file 11701_2026_3360_MOESM3_ESM.docx]

**Table 1.** GRADE assessment of certainty of evidence for outcomes in the meta-analysis comparing robotic and laparoscopic living donor hepatectomy.

| **Certainty assessment** | | | | | | | **№ of patients** | | **Effect** | | **Certainty** | **Importance** |
| --- | --- | --- | --- | --- | --- | --- | --- | --- | --- | --- | --- | --- |
| **№ of studies** | **Study design** | **Risk of bias** | **Inconsistency** | **Indirectness** | **Imprecision** | **Other considerations** | **robotic** | **laparoscopic** | **Relative (95% CI)** | **Absolute (95% CI)** |  |  |
| **Operative Time** | | | | | | | | | | | | |
| 6 | non-randomised studies | serious^a^ | very serious^b^ | not serious | not serious | none | 1862 | 757 | - | MD **87.26 higher** (50.89 higher to 123.62 higher) | ⨁◯◯◯ Very low^a,b^ | IMPORTANT |
| **Estimated Blood Loss** | | | | | | | | | | | | |
| 6 | non-randomised studies | serious^a^ | serious^c^ | not serious | not serious | none | 1862 | 757 | - | MD **111.48 lower** (147.14 lower to 75.83 lower) | ⨁⨁◯◯ Low^a,c^ | IMPORTANT |
| **Conversion to Open Hepatectomy** | | | | | | | | | | | | |
| 6 | non-randomised studies | serious^a^ | not serious | not serious | not serious | none | 38/1862 (2.0%) | 46/757 (6.1%) | **RR 0.42** (0.11 to 1.56) | **35 fewer per 1,000** (from 54 fewer to 34 more) | ⨁⨁⨁◯ Moderate^a^ | CRITICAL |
| **Blood transfusion** | | | | | | | | | | | | |
| 5 | non-randomised studies | serious^a^ | not serious | not serious | not serious | none | 4/949 (0.4%) | 14/592 (2.4%) | **RR 0.27** (0.09 to 0.76) | **17 fewer per 1,000** (from 22 fewer to 6 fewer) | ⨁⨁⨁◯ Moderate^a^ | CRITICAL |
| **Overall Vascular Complications** | | | | | | | | | | | | |
| 4 | non-randomised studies | serious^a^ | not serious | not serious | not serious | none | 72/797 (9.0%) | 27/294 (9.2%) | **RR 1.14** (0.71 to 1.83) | **13 more per 1,000** (from 27 fewer to 76 more) | ⨁⨁⨁◯ Moderate^a^ | CRITICAL |
| **Hepatic Artery Thrombosis (HAT)** | | | | | | | | | | | | |
| 3 | non-randomised studies | serious^a^ | not serious | not serious | serious^d^ | none | 34/674 (5.0%) | 8/204 (3.9%) | **RR 0.85** (0.36 to 2.00) | **6 fewer per 1,000** (from 25 fewer to 39 more) | ⨁⨁◯◯ Low^a,d^ | CRITICAL |
| **Reoperation rate** | | | | | | | | | | | | |
| 2 | non-randomised studies | not serious | not serious | not serious | very serious^d^ | none | 2/121 (1.7%) | 1/121 (0.8%) | **RR 1.36** (0.10 to 19.27) | **3 more per 1,000** (from 7 fewer to 151 more) | ⨁⨁◯◯ Low^d^ | CRITICAL |
| **Comprehensive Complication Index (CCI)** | | | | | | | | | | | | |
| 2 | non-randomised studies | serious^a^ | not serious | not serious | not serious | none | 624 | 154 | - | MD **8.55 lower** (14.95 lower to 2.16 lower) | ⨁⨁⨁◯ Moderate^a^ | IMPORTANT |
| **Mortality rate** | | | | | | | | | | | | |
| 5 | non-randomised studies | serious^a^ | not serious | not serious | not serious | none | 50/847 (5.9%) | 12/344 (3.5%) | **RR 0.99** (0.50 to 1.98) | **0 fewer per 1,000** (from 17 fewer to 34 more) | ⨁⨁⨁◯ Moderate^a^ | CRITICAL |
| **Recipient Hospital Stay (Days)** | | | | | | | | | | | | |
| 5 | non-randomised studies | serious^a^ | serious^c^ | not serious | not serious | none | 847 | 344 | - | MD **3.06 lower** (9.9 lower to 3.78 higher) | ⨁⨁◯◯ Low^a,c^ | IMPORTANT |
| **Donor Hospital Stay (Days)** | | | | | | | | | | | | |
| 6 | non-randomised studies | serious^a^ | very serious^b^ | not serious | not serious | none | 1862 | 757 | - | MD **0.14 lower** (1.08 lower to 0.79 higher) | ⨁◯◯◯ Very low^a,b^ | IMPORTANT |
| **Recipient Infection Rate** | | | | | | | | | | | | |
| 2 | non-randomised studies | not serious | not serious | not serious | not serious | none | 88/622 (14.1%) | 11/86 (12.8%) | **RR 0.60** (0.20 to 1.76) | **51 fewer per 1,000** (from 102 fewer to 97 more) | ⨁⨁⨁⨁ High | IMPORTANT |
| **Donor Infection Rate** | | | | | | | | | | | | |
| 3 | non-randomised studies | serious^a^ | serious^c^ | not serious | serious^d^ | none | 7/1781 (0.4%) | 7/1886 (0.4%) | **RR 0.41** (0.06 to 2.99) | **2 fewer per 1,000** (from 3 fewer to 7 more) | ⨁◯◯◯ Very low^a,c,d^ | IMPORTANT |
| **Recipient Overall Biliary Complications** | | | | | | | | | | | | |
| 5 | non-randomised studies | serious^a^ | not serious | not serious | not serious | none | 89/847 (10.5%) | 74/344 (21.5%) | **RR 0.62** (0.45 to 0.86) | **82 fewer per 1,000** (from 118 fewer to 30 fewer) | ⨁⨁⨁◯ Moderate^a^ | CRITICAL |
| **Donor Overall Biliary Complications** | | | | | | | | | | | | |
| 2 | non-randomised studies | serious^a^ | serious^c^ | not serious | serious^d^ | none | 7/868 (0.8%) | 11/1721 (0.6%) | **RR 0.85** (0.08 to 9.24) | **1 fewer per 1,000** (from 6 fewer to 53 more) | ⨁◯◯◯ Very low^a,c,d^ | CRITICAL |
| **Recipient Biliary Leakage** | | | | | | | | | | | | |
| 2 | non-randomised studies | not serious | not serious | not serious | not serious | none | 7/121 (5.8%) | 11/121 (9.1%) | **RR 0.64** (0.25 to 1.59) | **33 fewer per 1,000** (from 68 fewer to 54 more) | ⨁⨁⨁⨁ High | CRITICAL |
| **Donor Biliary Leakage** | | | | | | | | | | | | |
| 3 | non-randomised studies | serious^a^ | serious^c^ | not serious | serious^d^ | none | 10/960 (1.0%) | 10/1813 (0.6%) | **RR 1.18** (0.28 to 4.93) | **1 more per 1,000** (from 4 fewer to 22 more) | ⨁◯◯◯ Very low^a,c,d^ | CRITICAL |
| **Recipient Overall Morbidity** | | | | | | | | | | | | |
| 2 | non-randomised studies | serious^a^ | not serious | not serious | not serious | none | 267/624 (42.8%) | 94/154 (61.0%) | **RR 0.69** (0.56 to 0.84) | **189 fewer per 1,000** (from 269 fewer to 98 fewer) | ⨁⨁⨁◯ Moderate^a^ | CRITICAL |
| **Donor Overall Morbidity** | | | | | | | | | | | | |
| 3 | non-randomised studies | serious^a^ | serious^c^ | not serious | not serious | none | 76/1597 (4.8%) | 86/525 (16.4%) | **RR 0.47** (0.25 to 0.89) | **87 fewer per 1,000** (from 123 fewer to 18 fewer) | ⨁⨁◯◯ Low^a,c^ | CRITICAL |
| **Recipient Major Morbidity** | | | | | | | | | | | | |
| 4 | non-randomised studies | serious^a^ | not serious | not serious | not serious | none | 195/797 (24.5%) | 132/294 (44.9%) | **RR 0.58** (0.47 to 0.71) | **189 fewer per 1,000** (from 238 fewer to 130 fewer) | ⨁⨁⨁◯ Moderate^a^ | CRITICAL |
| **Donor Major Morbidity** | | | | | | | | | | | | |
| 7 | non-randomised studies | very serious^e^ | serious^c^ | not serious | serious^d^ | none | 26/2098 (1.2%) | 36/2236 (1.6%) | **RR 1.21** (0.42 to 3.47) | **3 more per 1,000** (from 9 fewer to 40 more) | ⨁◯◯◯ Very low^c,d,e^ | CRITICAL |
| **First Warm Ischemia Time (Minutes)** | | | | | | | | | | | | |
| 3 | non-randomised studies | not serious | very serious^b^ | not serious | not serious | none | 261 | 232 | - | MD **5.15 higher** (3.07 lower to 13.38 higher) | ⨁⨁◯◯ Low^b^ | IMPORTANT |
| **Second Warm Ischemia Time (Minutes)** | | | | | | | | | | | | |
| 2 | non-randomised studies | not serious | serious^c^ | not serious | not serious | none | 622 | 86 | - | MD **7.29 higher** (2.24 higher to 12.35 higher) | ⨁⨁⨁◯ Moderate^c^ | IMPORTANT |
| **Cold Ischemia Time (Minutes)** | | | | | | | | | | | | |
| 2 | non-randomised studies | not serious | not serious | not serious | not serious | none | 622 | 86 | - | MD **7.74 higher** (2.41 lower to 17.88 higher) | ⨁⨁⨁⨁ High | IMPORTANT |
| **Graft-out Time (Minutes)** | | | | | | | | | | | | |
| 3 | non-randomised studies | serious^a^ | very serious^b^ | not serious | serious^d^ | none | 225 | 258 | - | MD **94.17 higher** (44.88 higher to 143.47 higher) | ⨁◯◯◯ Very low^a,b,d^ | NOT IMPORTANT |

**CI:** confidence interval; **MD:** mean difference; **RR:** risk ratio

#### Explanations

a. one study show high risk of bias

b. Extremly high heterogeneity

c. High heterogeneity

d. Wide Confidence Interval

e. More than one study show high risk of bias
